# Specific MHC-I Peptides Are Induced Using PROTACs

**DOI:** 10.3389/fimmu.2018.02697

**Published:** 2018-11-22

**Authors:** Stephanie M. Jensen, Gregory K. Potts, Damien B. Ready, Melanie J. Patterson

**Affiliations:** Discovery Chemistry and Technology, AbbVie North Chicago, IL, United States

**Keywords:** PROTAC, MHC-I, HLA, immunopeptides, BET

## Abstract

Peptides presented by the class-I major histocompatibility complex (MHC-I) are important targets for immunotherapy. The identification of these peptide targets greatly facilitates the generation of T-cell-based therapeutics. Herein, we report the capability of proteolysis targeting chimera (PROTAC) compounds to induce the presentation of specific MHC class-I peptides derived from endogenous cellular proteins. Using LC-MS/MS, we identified several BET-derived MHC-I peptides induced by treatment with three BET-directed PROTAC compounds. To understand our ability to tune this process, we measured the relative rate of presentation of these peptides under varying treatment conditions using label-free mass spectrometry quantification. We found that the rate of peptide presentation reflected the rate of protein degradation, indicating a direct relationship between PROTAC treatment and peptide presentation. We additionally analyzed the effect of PROTAC treatment on the entire immunopeptidome and found many new peptides that were displayed in a PROTAC-specific fashion: we determined that these identifications map to the BET pathway, as well as, potential off-target or unique-to-PROTAC pathways. This work represents the first evidence of the use of PROTAC compounds to induce the presentation of MHC-I peptides from endogenous cellular proteins, highlighting the capability of PROTAC compounds for the discovery and generation of new targets for immunotherapy.

## Introduction

Peptides that are presented to the immune system by the major histocompatibility complex (MHC) serve as biomarkers that reflect the health of the parent cell. Peptide MHC class-I complexes are recognized by CD8^+^ T-cells through T-cell receptors (TCRs) and are essential to a properly functioning adaptive immune response. These peptides create unique three dimensional epitope surfaces when complexed with specific MHC proteins and serve as antigens for cellular disease, including both infection and cancer (1, 2). MHC-I peptides have become of particular interest as a source of novel targets for immunological therapies including chimeric antigen receptor-transduced T-cells (CAR-T), soluble T-cell receptors (sTCR), and peptide vaccines (3).

MHC class I peptides are derived from the proteolytic degradation of self-proteins, (so called retirees), as well as, defective ribosomal products (DRiPs) (4, 5). While there are some exceptions, protein sources of MHC-I peptides are generally processed through the proteasome in a ubiquitin-dependent fashion (6–8). Once generated, these peptides are transported into the endoplasmic reticulum (ER) by the transporter for antigen processing (TAP), trimmed by the N-terminal aminopeptidases ERAP1 and ERAP2, and loaded onto MHC class I proteins for transport to the cell surface and surveillance by cytotoxic CD8+ T-cells (9).

**Figure 1:**
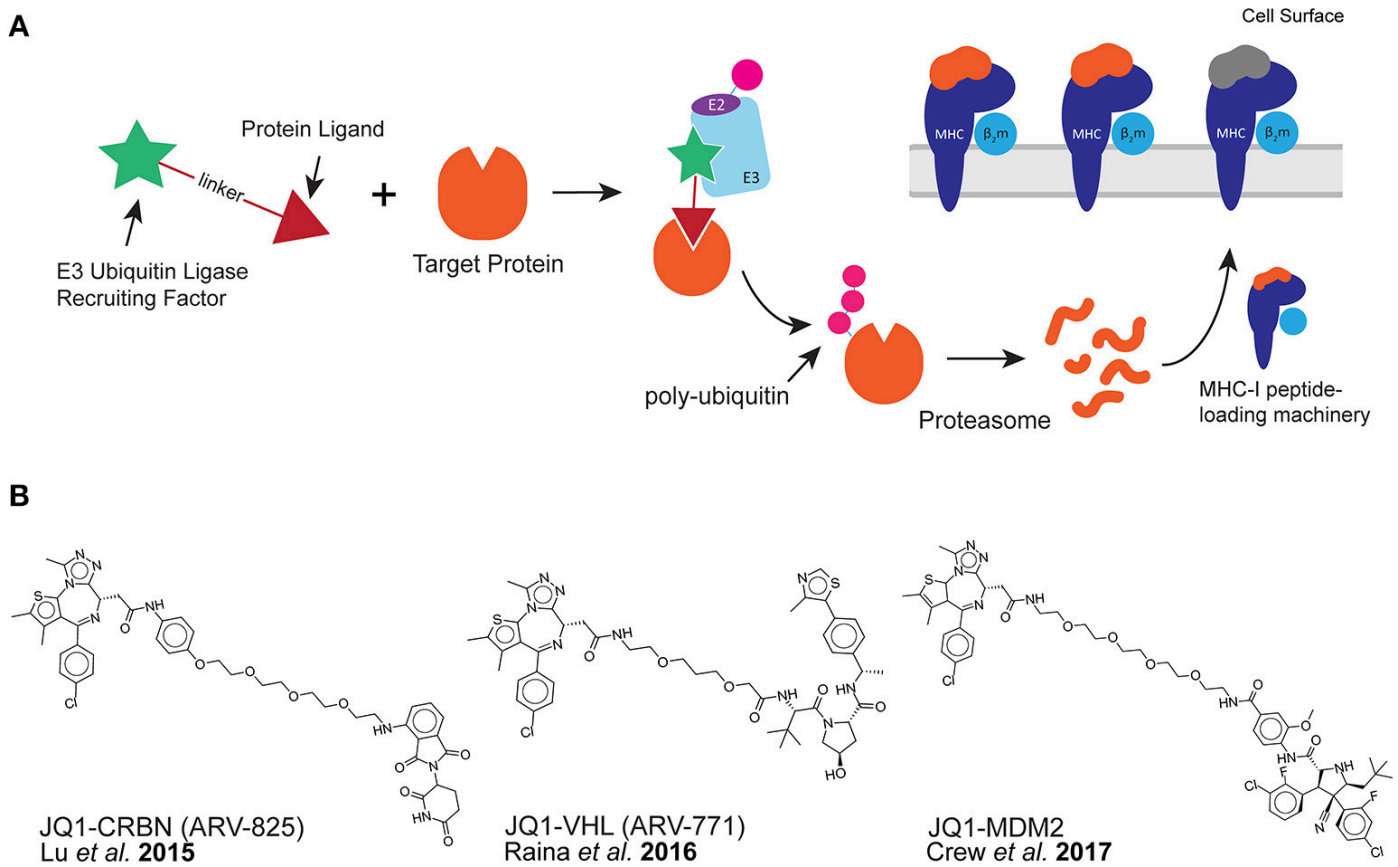
PROTAC compounds induce the presentation of MHC-I peptides. **(A)** PROTAC compounds consist of a protein ligand (triangle) and an E3 ubiquitin-ligase recruiting factor (star). PROTAC compounds induce the proteolytic degradation of target cellular proteins through a ubiquitin-mediated (pink circle) pathway. This could result in the presentation of MHC-I peptides derived from target protein. **(B)** PROTAC compounds used in this study: all three target the BET family of proteins through the small molecule JQ1 while recruiting different E3 ubiquitin-ligases.

Because the majority of peptides presented by MHC class I are thought to be derived from proteolytic degradation, we hypothesized that it might be possible to stimulate the presentation of specific MHC-I peptides through purposeful protein degradation. Recent developments in chemical biology have provided tools called proteolysis targeting chimera (PROTAC) with which to test our capability to do this (10). PROTAC compounds target specific proteins for proteasomal degradation through the recruitment of E3 ubiquitin ligases to a protein of interest. This is accomplished using a bi-functional chemical tool containing a protein-specific ligand and an E3 ubiquitin-ligase recruiting factor (Figure 1A) (10–12).

Protein degrading compounds were originally developed as an alternative to protein inhibitors: instead of binding to a protein and inhibiting activity, (while generally maintaining the cellular concentration of the target), PROTAC compounds act catalytically within a cell perpetually degrading the offending protein (13). We hypothesized that peptide products of PROTAC-induced degradation could be used for MHC-I antigen processing and presentation. If so, then we would be able to observe changes to the MHC-I peptide repertoire on the cell surface upon use of a PROTAC. Specifically, we expected to observe MHC-I peptides derived from PROTAC-targeted proteins.

Using a PROTAC with the intent of generating new MHC-I peptides on the cell surface could be advantageous for a few reasons. First, using a PROTAC compound could allow for the empirical detection of peptides from an endogenous protein of interest within a desired system. Currently, the field relies prediction algorithms to identify MHC-I peptides that might be produced from a protein of interest (14). Using a PROTAC would reduce the dependence upon predictions, which sometimes offer close, but not exact, presented peptide sequences. Additionally, induced MHC-I peptides could enhance the presence of certain peptides to make them more easily detected with available analytical methods.

A second advantage to using PROTACs as a catalyst for MHC peptide presentation would be the ability to induce or enhance specific peptides that could be targeted by T-cell based therapeutics. The ability to enhance MHC-I signals through general upregulation of the complex has already been shown to enhance T-cell responses (15). The ability to enhance a specific T-cell target in an endogenous system could provide a great advantage to the generation and application of T-cell based therapeutics.

Finally, the use of a PROTAC compound in the context of MHC-I presentation, provides the user with the ability to explore discrete components of the MHC-I presentation pathway by monitoring the degradation and processing of a protein of choice in an endogenous system, enabling increased biological understanding. Using mass spectrometry to more globally profile changes in the peptide repertoire upon PROTAC treatment opens up the possibility to explore not only components of the MHC-I presentation pathway but potentially unrealized biological pathways that are perturbed by directed protein target degradation.

Recently, a dTAG-fusion protein which contained the well-studied ovalbumin peptide SIINFEKL (S8L) was reported for use in a targeted degradation system. The S8L peptide was shown by flow cytometry to be presented by MHC-I upon degradation of the transfected source protein (16). While this study established a relationship between a target protein's intracellular concentration and its peptide presentation level, the use of PROTAC compounds to modulate the degradation of endogenous proteins has not been previously explored. Furthermore, no analyses on the effects of PROTAC treatment on the overall composition of the cellular immunopeptidome have been performed.

Herein, we demonstrate the use of PROTACs that successfully degrade their targets and induce the presentation of specific MHC-I peptides on the cell surface. We chose to use well-characterized bromo- and extra- terminal domain (BET) protein-directed PROTAC compounds (Figure 1B) to evaluate the effect of targeted protein degradation on MHC-I peptide presentation. These PROTACs were directed toward BET proteins using the small molecule bromo-domain-binder, JQ1 (17) while engaging distinct, orthogonal E3-ligase recruiting factors. Three PROTAC compounds were used in this study: JQ1-CRBN (also known as ARV-825) recruits a ligand for the E3 ligase cereblon (CRBN) using pomalidomide (18), JQ1-VHL (also known as ARV-771) recruits the von Hippel-Lindau (VHL) ligase (19), and finally, JQ1-MDM2 which recruits mouse double minute homolog 2 (MDM2) (20). Because some pairings of target protein to E3 ligase have been found to be more productive than others due to the conformational preferences of the ligase-target interaction (21–23), we were interested in comparing a range of PROTAC productivities on the presentation of MHC-I peptides. Furthermore, BET proteins are important disease targets due to their ability to recognize and bind acetylated lysine residues. In doing so, this protein family regulates transcriptional activity (24), and subsequently, BET proteins are validated targets for a number of indications in oncology (25, 26), Therefore, BET-specific PROTAC compounds have been gaining ground as a means for disease intervention (19, 27).

We additionally chose to examine the presentation of MHC-I peptides using JQ1-based PROTAC compounds because they have been extensively studied and offered a pre-defined system for assessing potential changes in complex mixtures of peptides potentially affected by treatment. In particular, structural studies performed with JQ1-VHL have revealed that cooperative binding between BRD proteins and E3-ligase proteins enable the selective degradation of BRD proteins (23). Furthermore, degradation is created through engagement of E3 ubiquitin ligases with specific bromodomains within the BRD protein structure. For instance, JQ1-VHL preferentially initiates degradation through binding to BRD4^BD2^>BD3^BD2^>BD2^BD1^ (23). For JQ1-MDM2 and JQ1-CRBN, these bromodomain-E3 ligase cooperative interactions have not yet been measured.

We utilized LC-MS/MS to identify isolated MHC-I peptides from PROTAC-treated and control-treated cells and found specific MHC-I peptides that were induced by PROTAC treatment. We looked at the relative abundance of these peptides over several time points to understand the relationship between protein degradation and presentation. We also examined changes in the entire observed immunopeptidome to understand global changes to MHC-I peptides caused by PROTAC treatment. Taken collectively, we have demonstrated a new method to induce the presentation of specific MHC-I peptide epitopes on the cell surface, effectively broadening the scope of targetable peptides that are displayed in a controlled manner.

## Methods

### Cell culture

BV173 cells were obtained from DSMZ (Cat#ACC-20, RRID:CVCL_0181) and cultured in 20% FBS, RPMI media (with 20 mM HEPES and L-glutamine; Sigma Aldrich, R7388) at 37°C, 5% CO_2_. BV173 cells were grown to a density of 1 × 10^6^ cells/mL prior to all treatments. For immunoprecipitation a total of 1 × 10^8^ cells were used for each experiment.

### Compounds and treatment

JQ1-CRBN, JQ1-VHL, and JQ1-MDM2 were all synthesized as previously described (18–20). JQ1 (SML0974) and pomalidomide (P0018) were purchased from Sigma-Aldrich. Treatments were performed in true biological triplicate. Cells were treated for FACS analysis (10 nM, 100 nM; 1–6 h), western blotting (0–1000 nM, 0–16 h), and immunoprecipitation of MHC-I complexes (0.1 −100 nM, 0–6 h). Initial immunoprecipitation-MS screening runs resulted in focused treatments: using PROTAC compounds at 10 nM, for 0–6 hours. All cellular treatments were at 37°C, 5% CO_2_ using BV173 cells at 1 × 10^6^ cells/mL density.

### Western blot

Primary antibodies anti-BRD2 (Bethyl, A302-583A), anti-BRD3, (Bethyl, A302-368A), and anti-BRD4 (Bethyl, A301-985A) were used at a 1:1000 dilution in Odyssey® Blocking Buffer (LI-COR, 927-40000). Anti-actin (Sigma-Aldrich, A2066) was used at a 1:5000 dilution. IRDye® 800CW goat anti-mouse (LI-COR, 926-32211) was used as secondary antibody at 1:5000 dilution. Blots were scanned using the LI-COR Odyssey® CLx scanner. For SDS-PAGE 30 μg of protein was used in each lane. SDS-PAGE-separated proteins were transferred to PVDF membranes for western blotting. Quantification of band intensity was performed in ImageJ using the area under the curve (AUC) from the protein of interest, normalized to actin AUC.

### Immunoprecipitation of MHC-I complexes

1 × 10^8^ BV173 cells were treated with JQ1, pomalidomide, JQ1-VHL, JQ1-MDM2, JQ1-CRBN, or DMSO. After treatment, cells were lysed in 2% CHAPS buffer (120 mM NaCl, 50 mM Tris-Cl pH 8.0, 2% CHAPS). Cells were ultracentrifuged at 100,000 × g for 1 h. Clarified supernatant was incubated with BB7.2 (BioLegend, 343302) for 1 h prior to incubation with protein A beads (Dynabeads™, Invitrogen,10008D) for 1 h. Beads were washed with 1% CHAPS buffer (120 mM NaCl, 50 mM Tris-Cl pH 8.0, 1% CHAPS) and 1X PBS. The remaining lysis supernatant was incubated with W6/32 (BioLegend, 311428) followed by a second incubation with protein A Dynabeads™. Complexes and peptides were eluted with 0.1% TFA prior to peptide separation using a 10 kDa cutoff spin filter (Pall Life Sciences, OD010C34). Peptides were desalted using C18 ziptips (Pierce, 87784) prior to injection onto MS instrumentation.

### LC-MS/MS method

Data-dependent acquisition of peptides was carried out using a Fusion Lumos mass spectrometer (Thermo Fisher Scientific) equipped with either an Easy nano LC 1200 system (Thermo Fisher Scientific) or Ultimate 3000 RSLCnano (Thermo Fisher Scientific). Mobile phases utilized for the Easy nano LC 1200 separation of peptides were 0.1% (v/v) formic acid (Thermo Fisher Scientific, Cat. No. 85170) in water (buffer A) and 0.1% (v/v) formic acid in acetonitrile (ACN) (Thermo Fisher Scientific, Cat. No. 85174) (buffer B). Samples were loaded onto an Acclaim PepMap 100 trap column (75 μm i.d. × 20 mm, Thermo Scientific, Cat. No. 164946) packed with 3 μm C18 resin and an Easy Spray analytical column (75 μm i.d. × 250 mm, Thermo Fisher Scientific, Cat. No.ES802) packed with 2 μm/100Å C18 resin. The column temperature was maintained at 45°C for the duration of each sample analysis. Peptides were eluted during a 95 min gradient with the following segments: an initial hold at 5% B for 2 min, 5–40% B for 80 min, and 40–100% B for 2 min, followed by an 11 min column wash and re-equilibration for 20 min at 5% B. For those samples analyzed using an Ultimate 3000 RSLCnano, the method incorporated additional loading buffers of 0.05% (v/v) TFA in water and 0.05% (v/v) TFA in ACN. The RSLCnano used an Acclaim PepMap 100 trap column (75 μm i.d. × 20 mm, Thermo Fisher Scientific, Cat. No. 164535) packed with 3 μm C18 resin. The remaining mobile phases and analytical column used with the RSLCnano were identical to the Easy nano LC 1200. Using the RSLCnano, peptides were eluted with a gradient comprised of the following segments: an initial hold at 2% B for 5 min, 2–40% B for 85 min, followed by a hold at 90% B for 10 min, and a return to 2% B with a final hold of 15 min. Despite the longer cumulative chromatographic separation, MS data was only acquired over 95 min to mimic the Easy nano LC 1200 separation. The Fusion Lumos full scan resolving power was set to 60,000 and acquired from 300 to 1750 *m/z*. An MS1 automated gain control (AGC) target of 4 × 10^5^ ions was used with a maximum injection time of 50 ms and a lock mass of 445.12 *m/z*. Peptides were selected for fragmentation if they fell between an intensity range from 1 × 10^4^-1 × 10^12^ and charge states +1–5. Peptides with +1 charge states were only sampled if they fell within a 750–1750 *m/z* range, while all +2–5 charge states were sampled. Each peptide fitting these criteria was sampled twice and then excluded from MS acquisition for 60 s within a 15 ppm precursor tolerance. Total MS cycle time was set to 5 s. Peptides were selected using a 1.2 *m/z* quadrupole isolation window and fragmented using collision-induced dissociation (CID) with 29% normalized collision energy. Resulting peptide fragments were analyzed with rapid scans in the ion trap mass analyzer using an AGC target of 3 × 10^3^ ions and a 35 ms maximum injection time.

### Search method

Peptide identifications were made using Byonic™ (Protein Metrics) software against the Human Uniprot database (downloaded on 8/10/2015) with a 20 ppm precursor mass tolerance, and 0.7 Da fragment ion mass tolerance. Peptide identifications were made with a 1% FDR. After compiling all identified peptides from all instrument runs, peptide identifications were filtered down to those made with +/– 5 ppm precursor mass tolerance. Furthermore, due to multiple discrete searches of the immunopeptidome raw files, the composite peptide list was searched using the NCBI BLAST algorithm (https://blast.ncbi.nlm.nih.gov) to determine consensus protein identifications across the data set. With these constraints applied, we further restricted the final peptide list to include peptides identified with >2 PSM.

### Label-free quantification

For label-free quantification of BET peptide abundance, 29 housekeeping peptides were selected for use in normalizing signals across data acquisitions. These peptides were shared across all samples and had a range of observed abundances within each sample. A small FASTA file containing only these housekeeping peptides and the observed BET peptides was created and used to search against all data files to accommodate import into Byologic (Protein Metrics™). Peptide identifications were manually verified in Byologic, and the extracted ion chromatogram area under the curve (XIC AUC) was generated for this subset of peptides as a measure of abundance. For each data file, normalization was performed using the average XIC AUC of the set of 29 housekeeping peptides. Normalized XIC AUC values for each peptide and condition were plotted as averages of all available observations +/– the standard deviation (SD). Single observations were annotated and assigned average experimental error (SD).

### Flow cytometry

BV173 cells were plated at a density of 1 × 10^6^ cells/mL and treated with 10 and 100 nM of each PROTAC compound (or DMSO) for 0, 1, 3, or 6 h. After treatment, cells were washed with FACS buffer (1X PBS, 1 mM EDTA, 1% FBS, 0.1% NaN_3_) and incubated with either BB7.2 or W6/32 using 1 μg antibody per 100 μL of cell suspension for 30 min at 4°C. Cells were washed with FACS buffer prior to incubation with secondary antibody (AlexaFluor488, donkey anti-mouse, Thermo Fisher, A-21202) at 4°C for 30 min. Cells were washed 2X with FACS buffer, prior to analysis on FACSCanto-II flow cytometer (BD Bioscience). FlowJo® software was used for data analysis. Cells were gated for live populations only. The median fluorescence intensity (MFI) was extracted and plotted for each sample as the average MFI +/– SD.

### Protein annotations and pathway mapping

Gene ontological annotations were assigned using the Panther classification system (pantherdb.org) (28). To determine overrepresentation of specific cellular components within our dataset against the entire human proteome we used Fisher's Exact with FDR multiple test correction, available as a component of the Panther system. Annotation visualization was performed with Proteomaps software, using the number of unique peptides for each source protein as a measure of significance (www.proteomaps.net) (29). Biological pathway mapping was performed using Ingenuity Pathway Analysis® (Qiagen). Core pathway analysis was performed with our dataset using only human experimentally-determined and high-confidence relationships.

### Quantification and statistical analysis

Statistical details of experiments can be found in figure legends. Unless otherwise stated, data is plotted as the mean +/– SD.

## Results

### PROTAC compounds degrade target proteins

JQ1-CRBN, JQ1-VHL, and JQ1-MDM2 were all synthesized as previously described (18–20) and assessed for the capability to degrade BET target proteins in BV173 cells by western blot (Figure S1). Degradation of BRD proteins by these JQ1-PROTACs was observed over a range of concentrations. Time-dependent degradation was also assessed to compare the relative rates of degradation of the BET proteins by each PROTAC within a focused concentration range (Figure S2). The efficiency of protein degradation was consistent with previous reports (18, 20, 30). JQ1-CRBN and JQ1-VHL performed similarly, successfully degrading BRD2, BRD3, and BRD4. However, JQ1-MDM2 was less effective at the degradation of BET proteins, overall.

### PROTAC treatment does not change the amount of MHC-I on the cell surface

**Figure 2:**
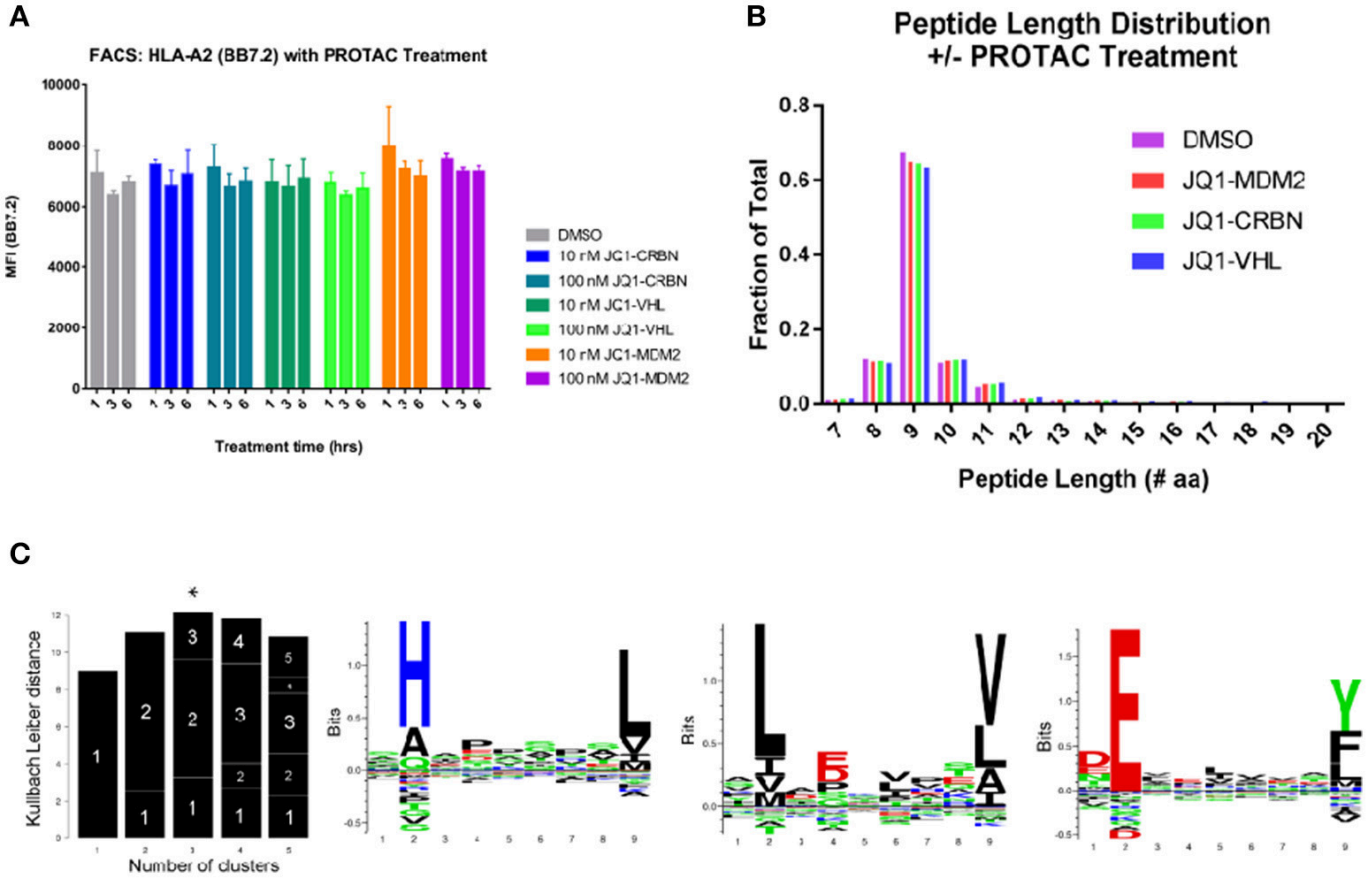
General effects of PROTAC treatment on BV173 cells. **(A)** PROTAC treatment does not alter the amount of MHC-I on the cell surface: analyzed by flow cytometry and HLA-A2 staining (BB7.2), as well as, W6/32 (pan MHC-I, Figure S3). **(B)** MHC-I peptide length distribution from PROTAC-treated and DMSO-treated cells: PROTAC treatment does not change the length distribution of identified MHC-I peptides as evaluated for each PROTAC across all treatment time points and concentrations (aggregate values represented for each condition, plotted as fraction of total). **(C)** Gibbs clustering analysis revealed three predominant motifs present within isolated and identified HLA peptides. HLA alleles present in our tested cell line: HLA-A^*^02:01, HLA-A^*^30:01, HLA-B^*^18:01, HLA-B^*^15:10, HLA-C^*^12:03, and HLA-C^*^03:04.

To understand the contributions of peptide changes to the total immunopeptide pool, we analyzed the effect of PROTAC treatment (at 10 and 100 nM from 0 to 6 h) on the amount of MHC-I on the cell surface using flow cytometry. We stained for the presence of MHC-I using pan-HLA antibody W6/32, as well as, HLA-A^*^02 antibody BB7.2 and found no increase in total MHC-I on the cell surface (Figure 2A, Figure S3) indicating that any changes in specific peptides observed were not due to upregulation of MHC-I itself.

### Isolated peptides possess similar HLA-binding characteristics with and without Protac treatment

We isolated MHC-I peptides from BV173 cells through immunoprecipitation of MHC-I complexes using both BB7.2 (anti-HLA-A2) and W6/32 (anti-pan-MHC-I) antibodies. Peptides eluted from MHC-I complexes were detected by LC-MS/MS, and the resulting spectra were interrogated using Byonic software (Protein Metrics™). Peptides were identified by searching against a human protein database (Uniprot), and the results were filtered to a 1% false discovery rate (FDR). To further standardize the data, peptide identifications that resulted from >5 ppm precursor ion mass accuracy and/or a single peptide spectral match (PSM) were eliminated. After applying these criteria, we retained approximately 7,500 unique peptide sequences from 3,200 protein sources in aggregate across all experiments. The complete list of identified peptides can be found in a supplemental file (Table S1). This prioritized list consisted of peptides ranging in size with the majority (92%) falling between 8 and 14 amino acids in length. We found that PROTAC treatment (or control compound treatment) had no effect on this length distribution within our dataset (Figure 2B). Additionally, across all samples, approximately 60% of identified peptides were 9-mers, which is consistent with previous reports of confident MHC-I isolation (31).

To aid in the conformation that the isolated peptides had features relevant to HLA-binding, we analyzed the motifs of our isolated immunopeptides and found that (1) these motifs were consistent between treated and untreated samples and (2) the identified motifs were appropriate for the HLA alleles present in the BV173 cell line (HLA-A^*^02:01, HLA-A^*^30:01, HLA-B^*^18:01, HLA-B^*^15:10, HLA-C^*^12:03, and HLA-C^*^03:04) (Figure 2C).

The affinity of isolated immunopeptides was predicted using the Immune Epitope Database (http://www.iedb.org/) (32) with the application of Stabilized Matrix Method (SMM) and Artificial Neural Networks (ANN) affinity algorithms to all identified peptide sequences between 8 and 14 amino acids (33–35). From our complete dataset, we observed increased representation of peptides with higher predicted affinity for HLA-A^*^02:01. However, we were still able to identify peptides with high predicted affinity for all represented MHC-I alleles within our cell line from those alleles that were available within the IEDB resource. We compared the affinity profile of PROTAC-treated samples to DMSO-treated samples using both SMM and ANN algorithms and found no changes in the distribution of high affinity (0–100 nM IC50) peptides with PROTAC treatment. However, we did observe a small increase in the amount of mid-range affinity (1–10 μM IC50) HLA-A^*^02 peptides with PROTAC treatment, with a corresponding decrease in HLA-B^*^18 representation within the same affinity range. This effect was observed upon analysis with both SMM and ANN affinity algorithms (Figure S4). Across all other represented alleles, we observed no changes to the distribution of predicted peptide affinity. In total, PROTAC-treated vs. untreated samples maintained the same presentation profile, with a small variance in peptide affinity at mid-range concentrations, in an allele-specific fashion.

### The ontological distribution of identifications was consistent for treated and untreated samples

The composition of the BV173 MHC-I peptidome was globally assessed using Panther and Proteomaps. The Panther classification system (36) was used for the assignment of gene ontology (GO) terms. Using this tool, we compared MHC-I peptides isolated from each treatment against the human proteome to look for overrepresentation of specific cellular components and biological processes (28). Consistent with previous reports of HLA peptide isolation (37, 38) we observed overrepresentation of peptides that were primarily derived from cellular components such as the protein-DNA complex (*p*-value = 7.54E-05), nucleus (*p*-value = 7.98E-47), and ribosome (*p*-value = 4.14E-10), with an underrepresentation of membrane-bound proteins (*p*-value = 4.29E-06) in all samples. Across all treated samples (all samples with the exception of DMSO) we observed an increase in glycolysis overrepresentation (*p*-value = 1.58E-04). With this exception, we did not identify any significant changes in gene ontology between PROTAC-treated and control samples (Table S2).We additionally used Proteomaps software for visualization of ontological distributions within our dataset (Figure S5) (29, 39). Using the number of unique peptides for each source protein as a measure of significance, our Proteomaps representation was similar to previous reports and was not found to vary between PROTAC-treated and control samples.

### BET (and other) source proteins were observed uniquely in PROTAC-treated samples

**Figure 3:**
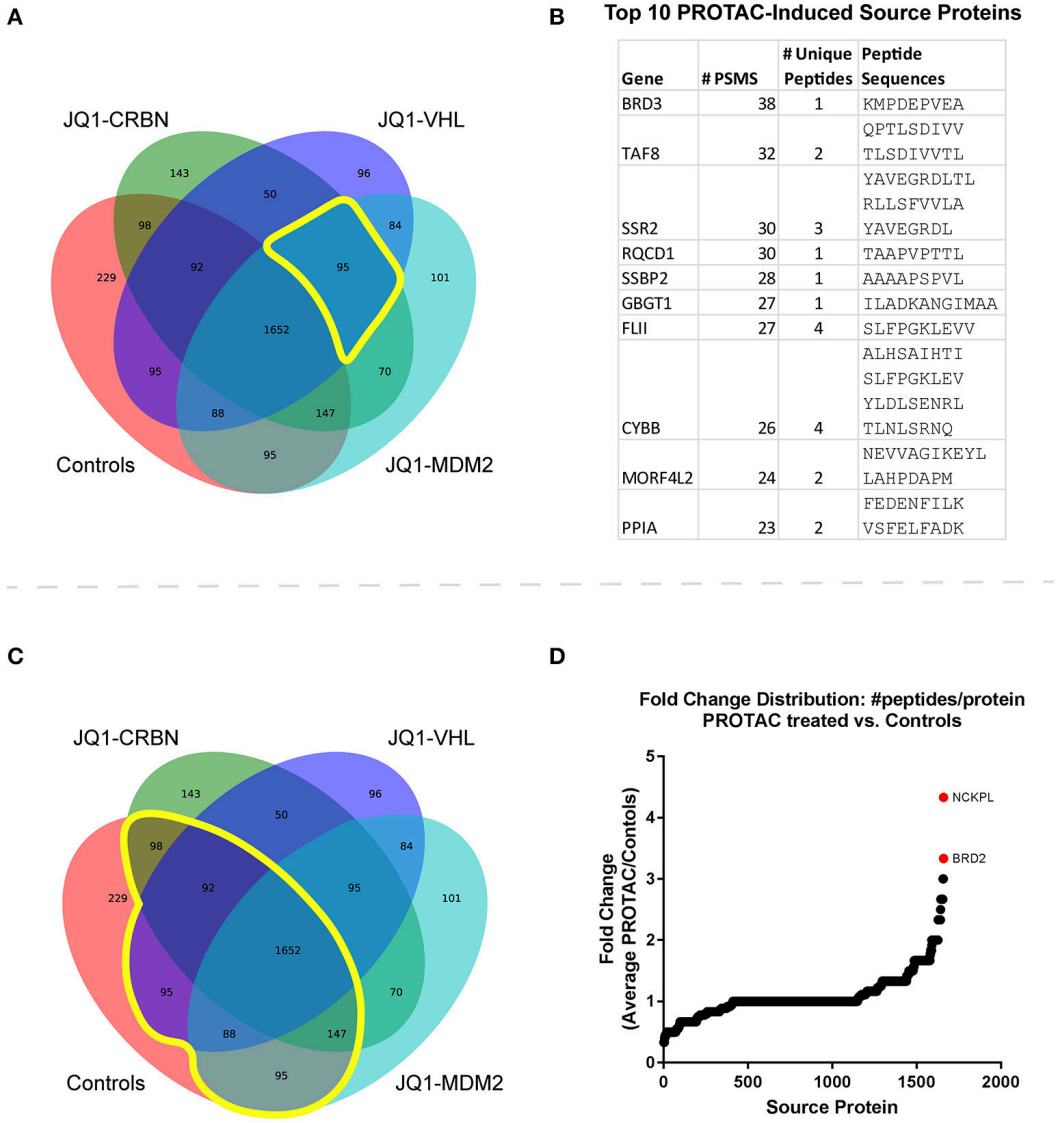
Immunopeptide source protein identification by treatment across all time points and concentrations. **(A)** Venn diagram of source protein identifications made across all samples (*n* = 166). Control treatments include JQ1, pomalidomide, and DMSO. There were 95 source proteins uniquely identified in all 3 PROTAC-treated samples. **(B)** The top ten identified PROTAC-specific proteins, ranked by number of observations (PSMs) with corresponding MHC-I peptide sequences. **(C)** Venn diagram of source protein identifications made across all samples. 1,652 source protein identifications were shared between treated and control samples. **(D)** Fold change was calculated for proteins present in both treated and control samples from the list of shared protein identifications. Changes in the number of unique peptides per protein were analyzed for each treatment vs. controls (DMSO, JQ1, and pomalidomide). Fold change = [peptides/protein]_PROTAC_/[peptides/protein]_Controls_. The average fold change across all PROTAC-treatments vs. controls was plotted and source proteins with the greatest increase in representation are highlighted. Fold change was calculated for proteins present in both treated and control samples.

To examine PROTAC-dependent changes to source protein identifications, we took two approaches. First, we looked at which source proteins were observed only upon PROTAC treatment, but not control treatment. There were 95 source proteins uniquely observed after treatment with all three JQ1-based PROTAC compounds (Figure 3A). We sought to rank these treatment-specific source protein identifications by using the number of PSMs per protein as a measure of prevalence. We were then able to rank the PROTAC-specific proteins from most-to-least likely to be induced by BET PROTAC treatment. Significantly, BRD3 (a BET target protein) ranked as having the most observed PSMs, followed closely by TAF8 (a transcription-related protein). The top 10 most-represented (by PSM count, across 166 replicates total, 122 PROTAC-treated replicates) PROTAC-specific protein identifications are listed in Figure 3B. The complete list of PROTAC-unique source protein identifications can be found in Table S3.

### PROTAC treatment enhanced source protein coverage for BET proteins and others

The second approach involved identification of source proteins for which some peptides were observed in controls (prior to treatment), but for which PROTAC-treatment enhanced their presentation through increased peptide coverage. We compared changes in the total number of peptides-per-protein between treated and untreated samples, hypothesizing that proteins already represented in control-treated samples might present additional peptides when stimulated by PROTAC treatment (Figure 3C). For this analysis, we used a ratio of ratios: the number of peptides/protein was calculated for each source protein within each treatment, and this value was compared for each protein between treatments: protein-specific fold change = [peptides/protein]_PROTAC_/[peptides/protein]_Controls_. From this analysis, we were able to determine specific proteins that had increased representation in PROTAC-treated samples vs. controls (Figure 3D). Significantly, BRD2, Nck-associated protein 1-like, (NCKPL) and a handful of other proteins showed increased representation after PROTAC treatment. The complete list of all source proteins and PROTAC-specific changes in source protein representation can be found in Table S4.

### BET peptide sequences were uniquely observed in PROTAC-treated samples

**Figure 4:**
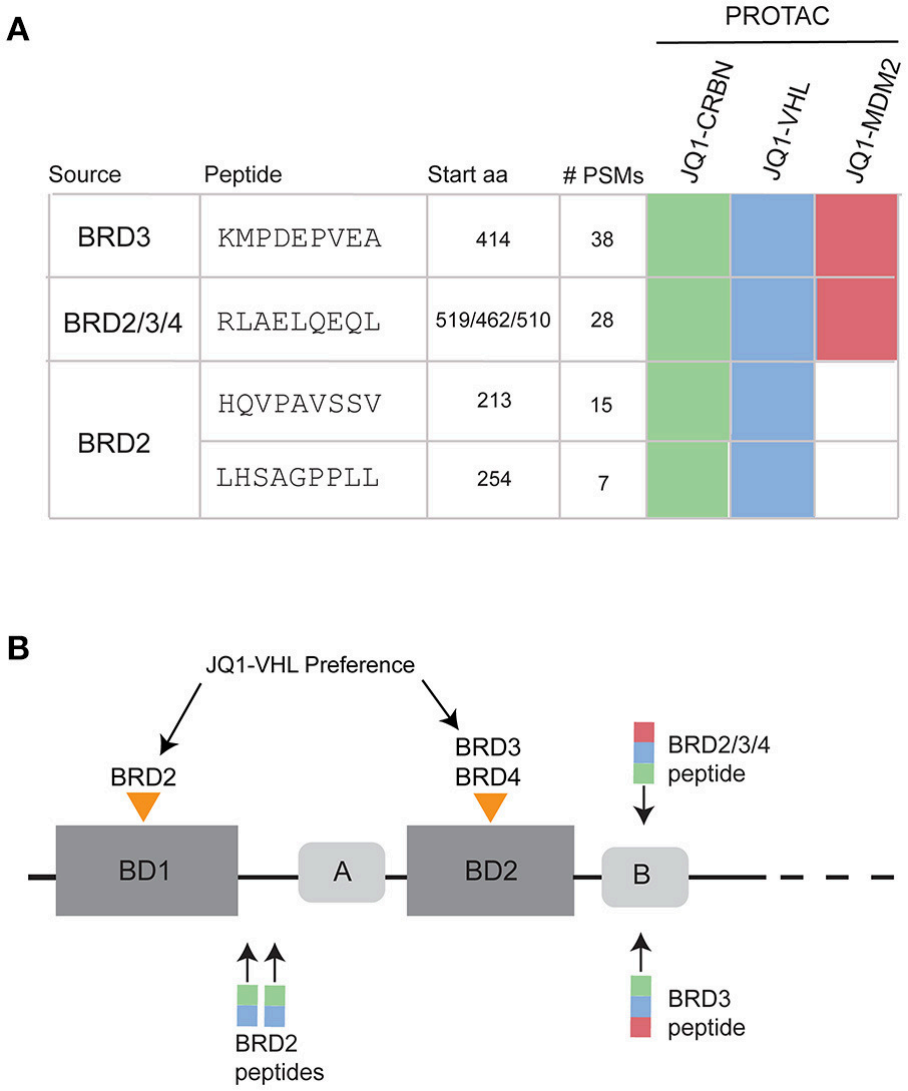
PROTAC-induced BRD peptides across all time points and concentrations. **(A)** MHC-I peptides observed uniquely after PROTAC treatment. Sequences were verified with synthetic spectra (Figure S6). Map of specific PROTAC compounds that induced presentation of each peptide. **(B)** BRD protein family domain map, showing the relative locations of bromodomain 1 (BD1) and bromodomain 2 (BD2) as well as two conserved regions (A and B) within the protein sequence. JQ1 cooperatively binds and degrades BRD2 within BD1. JQ1-VHL cooperatively binds and degrades BRD3 and BRD4 within BD2. Observed peptides derived from BRD2, BRD3, and BRD2/3/4 are annotated over the BRD family protein structure, with colored boxes representing JQ1-CRBN (green), JQ1-VHL (blue), and JQ1-MDM2 (red) induced presentation.

BET-PROTAC compounds induced the presentation of BET-derived MHC-I peptides. PROTAC-specific sequences that we could verify with synthetic peptide MS/MS spectra (Figure S6) and with >2 PSM are listed in Figure 4A. These peptides were not observed in samples prepared from JQ1-, pomalidomide-, or DMSO-treated cells. To verify the uniqueness of these peptides to treatment, the ion chromatograms for the precursor masses (+/– 10 ppm) were extracted and compared across samples. An example for BRD3-specific peptide is shown in Figure S7. We found no evidence of the presentation of these peptides in control samples within the limits of our detection.

When looking at the list of PROTAC-induced BET peptides (Figure 4A), we observed that all three JQ1-PROTAC compounds induced the same two MHC-I peptides: KMDPEVEA (BRD3) and RLAELQEQL, (a peptide originating from a conserved region of the BRD2/3/4 proteins). These two peptides were observed, regardless of E3-ligase recruiting factor, and not in controls. Additionally, JQ1-CRBN and JQ1-VHL both induced the presentation of HQVPAVSSV and LHSAGPPLL from BRD2. We did not observe BRD2 peptides from JQ1-MDM2 treatment. We also noticed that for each BRD protein, PROTAC-induced peptide presentation occurred in close proximity to either the first (BRD2) or second (BRD3, BRD2/3/4) bromodomain (BD) of each protein (Figure 4B).

**Figure 5:**
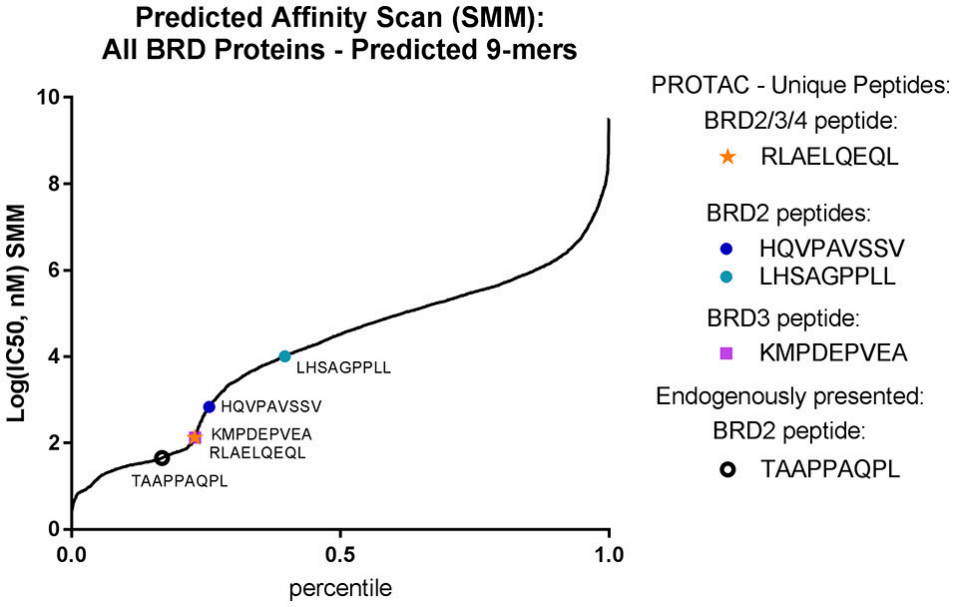
PROTAC-induced BRD peptides have sub-optimal affinity for BV173 alleles. Identified PROTAC-induced BRD peptides, mapped over predicted affinity of all potential BRD2 and BRD3 9-mers (IC50, nM) predicted with the SMM algorithm.

We analyzed the predicted MHC-I binding affinity for the four identified BRD peptides using both SMM and ANN algorithms and found that they had a range of predicted IC50 values for appropriate alleles. However, no newly detected peptide was predicted to bind with better than 100 nM (IC50) affinity (Figure 5). When the complete sequence of each BRD protein was scanned for predicted HLA-binding 9-mers using the IEDB affinity resource, we noticed that there were several peptide candidates which were predicted to be stronger binders for available MHC-I alleles. These peptide sequences were not selected by the cell for presentation within our limits of detection. Additionally, the endogenously presented BRD2 peptide TAAPPAQPL present in both controls and treated samples had a higher predicted affinity for MHC-I than any of the peptides induced with treatment.

### The abundance of BET-derived immunopeptides was dependent on PROTAC concentration and treatment time

**Figure 6:**
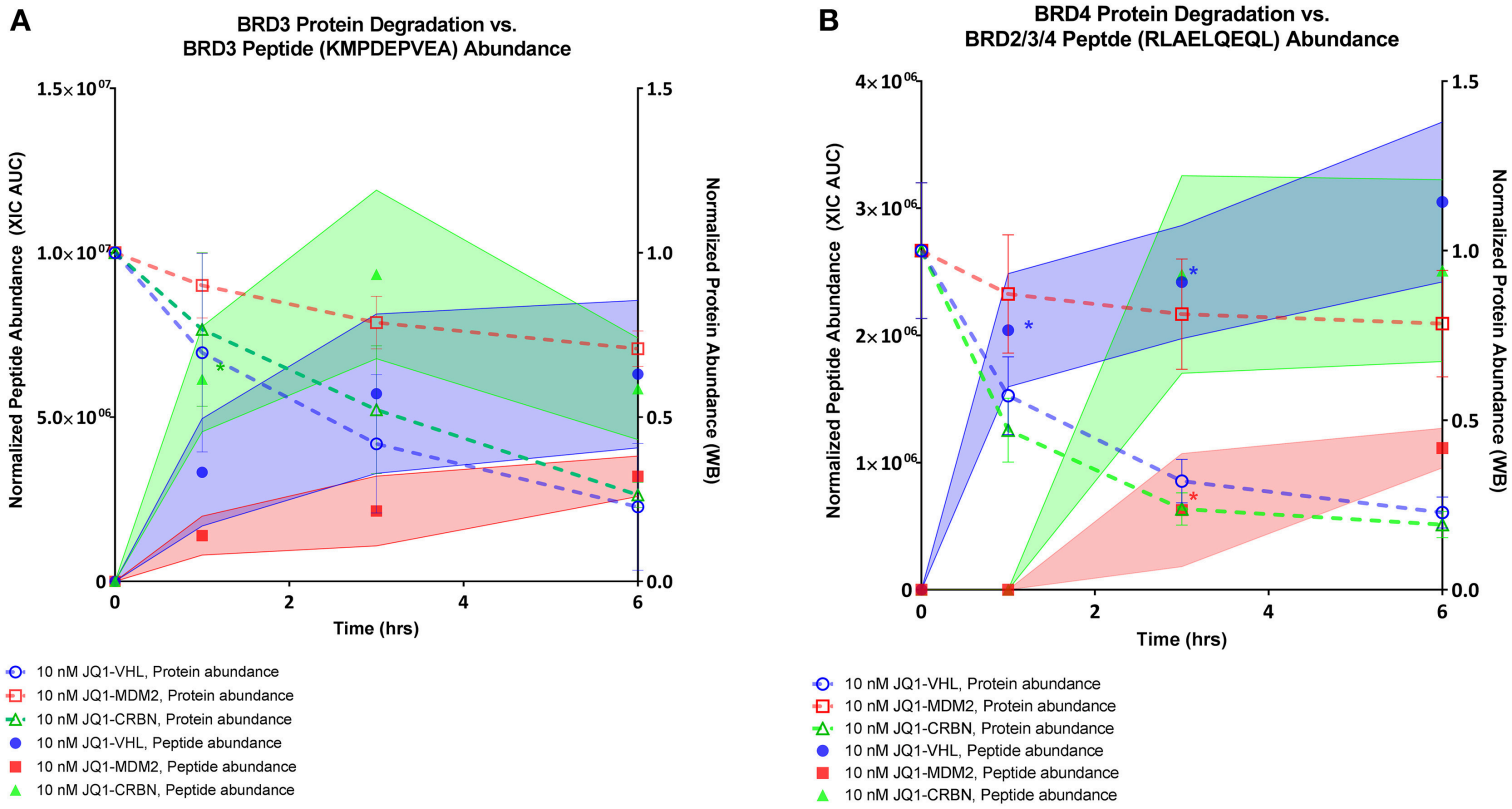
BRD peptide presentation, 10 nM PROTAC compounds. **(A)** Relative abundance of BRD3 peptide KMPDEPVEA over a 6 h time course across all 3 PROTAC treatments overlaid with relative abundance of BRD3 protein as assessed by western blot. **(B)** Relative abundance of BRD2/3/4 peptide RLAELQEQL over a 6 h time course across all 3 PROTAC treatments overlaid with relative abundance of BRD4 protein as assessed by western blot. Shaded regions of peptide abundance curves are standard deviation over multiple replicates (2–6 replicates). Peptide abundance data (XIC AUC) and exact number of replicates is supplied in Table S5. Western blot data is plotted as the mean over three replicates, +/– SD. ^*^indicates single observation, with average experimental error applied.

To compare the relative abundances of isolated immunopeptides across different conditions, a label-free MS quantification method was used. In order to normalize signals across all samples, we applied correction factors based on a set of 29 housekeeping peptides, selected due to their consistent representation and range of abundances (Table S6, Figure S8). We measured relative abundances of the two most frequently observed BET peptides, KMDPEVEA, and RLAELQEQL, over a 6 h time course oftreatment with 10 nM of each PROTAC compound (Figure 6). PROTAC-induced BRD2 peptides were not present prevalently enough in our dataset to plot abundance curves. Their sporadic occurrence was consistent with less-robust degradation of BRD2 protein. When comparing the relative abundance of PROTAC-induced peptides over a 6 h time course, we observed that JQ1-VHL and JQ1-CRBN were more efficient than JQ1-MDM2 at producing BET-derived peptides. The corresponding peptides were presented faster and reached maximum abundance within a shorter time frame when the former two PROTAC compounds were applied. We overlaid source protein degradation (measured by western blot, Figure S2) with each peptide abundance plot to compare the rate of degradation with the rate of peptide presentation. We observed that induced peptide abundance reflected source protein degradation in a PROTAC-specific fashion.

**Figure 7:**
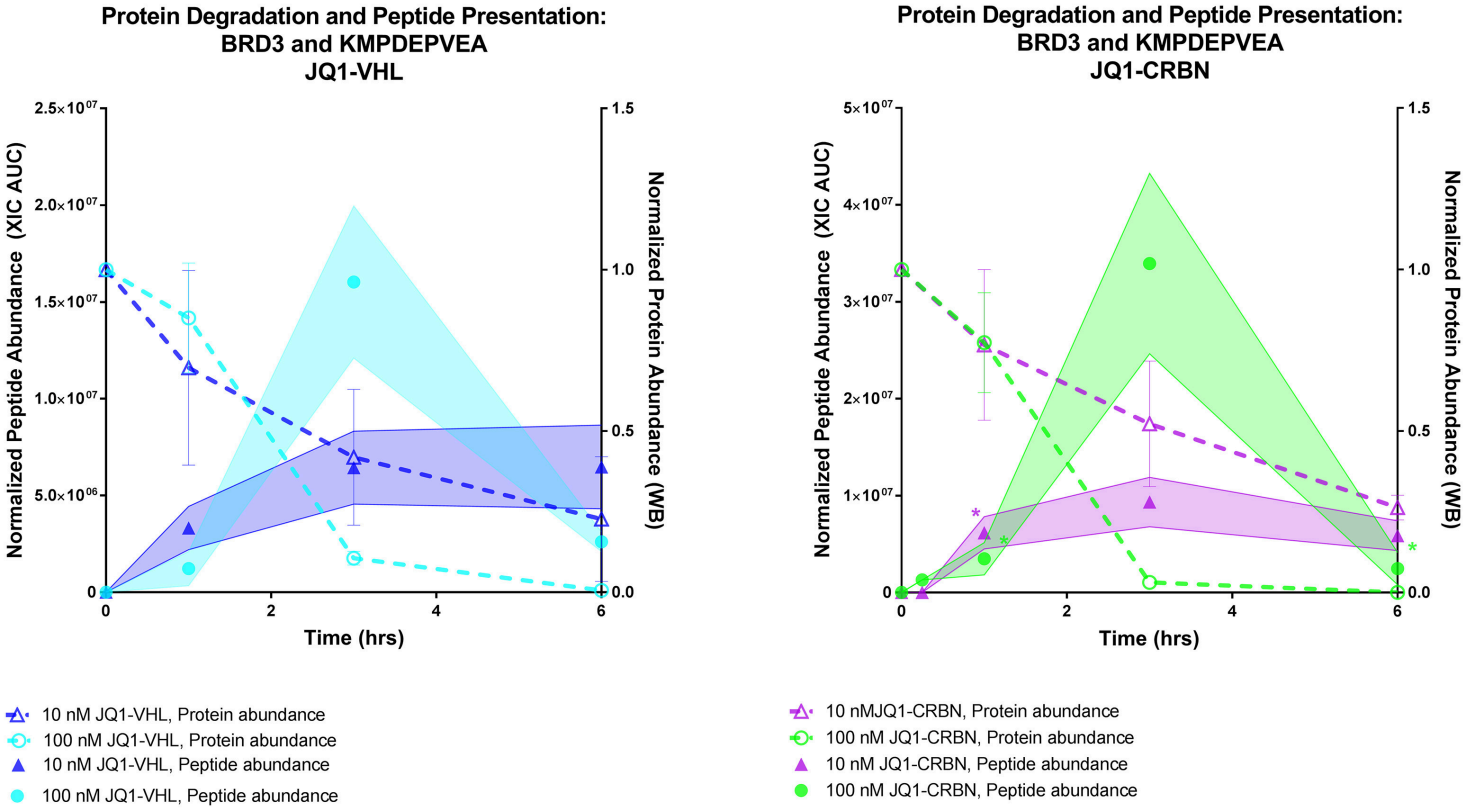
Comparing protein degradation and peptide presentation for BRD3 using different PROTAC concentrations. BRD3 protein degradation (Right Y axis, both plots) was measured by western blot.Protein degradation was measured using either 10 nM or 100 nM of either JQ1-VHL or JQ1-CRBN treatment over a 6 h time course. BRD3 peptide KMPDEPVEA presentation (Left Y axis) after treatment with10 or 100 nM JQ1-VHL or JQ1-CRBN treatment is shown. Peptide abundance data (XIC AUC) and number of replicates is supplied in Table S5. ^*^indicates single observation, with average experimental error applied.

We compared the rate of presentation of the BRD3 peptide KMDPEVEA between high (100 nM) and low (10 nM) concentrations of JQ1-VHL (left) and JQ1-CRBN (right) (Figure 7).We additionally compared this presentation to its corresponding source protein degradation (as observed by western blot). In doing so we observed several trends. First, a higher concentration of PROTAC induced a higher response within a 3 h time frame. Second, faster depletion of source protein at higher concentrations resulted in a depletion of presented peptide at later time points, and again, the overall rate of presentation of these peptides reflected the overall rate of degradation of protein induced by each PROTAC.

### PROTAC-induced pathway changes are observed within the detected HLA ligandome

In order to gain insight into potential unanticipated PROTAC-induced changes, we performed Ingenuity Pathway Analysis (R) (IPA) mapping of proteins that were uniquely represented in our PROTAC-treated dataset, as well as, those proteins that were shown to have increased coverage after PROTAC treatment. After performing a core mapping analysis with only high confidence or experimentally observed associations, we identified the mTOR pathway as being upregulated by PROTAC treatment (*p*-value = 5.07E-03), as well as, other closely associated pathways. A map of the represented pathway networks and associated *p*-values is located in Figure S9. Additionally, Myc (*p*-value = 1.29E-02), CDK4/6 (*p*-value = 6.32E-04), E2F1 (*p-*value = 7.82E-04), and E2F4 (*p-*value = 1.16E-03) were observed to be upstream transcriptional regulators of both PROTAC-unique and PROTAC-enhanced identifications in our dataset. A list of predicted upstream regulators of identifications made within our PROTAC-specific dataset is shown in Figure S10.

## Discussion

Taken collectively, the results of this study enabled the identification of both local and global changes to the MHC-I immunopeptidome after treatment with three BET-directed PROTAC compounds. We found that BET-PROTAC treatment induces the presentation of specific MHC-I peptides from target BRD proteins, as well as, peptides from other proteins that could be mapped to known pathways associated with BET inhibition and degradation. Despite these changes, we found that PROTAC treatment had no appreciable effect on the total amount of MHC-I on the cell surface. Furthermore, we found no major changes to the length, motif, or ontological distribution of observed peptide samples from any treatment condition (e.g., PROTAC, JQ1, pomalidomide, or DMSO). The lack of broad cellular perturbation observed is consistent with what one might expect with a pharmacologically-optimized ligand such as JQ1. However, we did see a small change in allele-specific peptide affinities at higher IC50 values, indicating that PROTAC treatment may change the cellular pool of available peptides for MHC-I processing and presentation. Others have noted that the amount of HLA protein available is the limiting component for peptide presentation (16, 40). Without stimulating cells to increase the abundance of MHC-I, peptides generated through induced proteolytic degradation compete for occupancy of MHC-I.

While other labs have emphasized the role and contribution of DRiPs to the HLA ligandome, the percentage of immunopeptides derived through this mechanism is contended (4, 41). Our assessment of the source of PROTAC-induced peptides is mainly based on kinetic observations. For instance, we found that the rate of BET peptide presentation matched the rate of targeted BET protein degradation. Also, protein degradation with higher concentrations of PROTACs rapidly depleted endogenous source protein, leading to the loss of detection of peptides derived from these proteins. Although it is still possible that some of the presented peptides induced by PROTAC treatment might be derived from DRiPs, our results suggest that the majority of changes in BET MHC-I peptide presentation were likely produced through stable protein ubiquitination and degradation.

In total, we detected four BRD–derived peptides that met our stringent criteria for identification upon treatment with BET-PROTAC compounds. These peptides were derived from BRD2, BRD3, and a conserved region shared between BRD2, BRD3, and BRD4 proteins (BRD2/3/4). It is possible that some of the BRD2/3/4 peptide RLAELQEQL was derived from endogenous BRD4 for multiple reasons. First, 10 nM of all PROTAC compounds only limitedly degraded BRD2 (Figure S2) resulting in the sporadic identification of BRD2 peptides across our time-course at this concentration, yet this peptide was robustly observed with 10 nM treatment within the same timeframe which would be consistent with either a BRD3 or BRD4 origin. Additionally, BRD2 was only limitedly degraded by JQ1-MDM2 within 6 h (Figure S2), yet JQ1-MDM2 treatment induced presentation of the peptide RLAELQEQL. We also observed that presentation of the BRD2/3/4 peptide was reflective of corresponding degradation of BRD4 (Figure 6B). And finally, kinetics of RLAELQEQL presentation were different than the BRD3-derived peptide KMPDEPVEA (despite similar source regions, and SMM predicted affinity for the same allele, HLA-A^*^02:01). However, predicted affinity alone is not sufficient to predict efficient processing through the MHC-I pathway, and these observations alone are not definitive in the designation of a source protein for RLAELQEQL.

We observed that all BET-derived peptides originated from distinct regions of each BRD protein. This observation could be due to both the specific proteasomal composition and the MHC-I processing machinery within our cell line, as others have found specific “hotspots” for antigen presentation within each protein (37). Interestingly, these peptide origin positions correspond well with reported cooperative binding preferences for JQ1-VHL within each protein. For instance, JQ1-VHL preferentially binds and degrades BRD4^BD2^> BRD3^BD2^> BRD2^BD1^(23), and this mapped well with the specific BD domains from which each peptide was derived, indicating that ligand binding to specific regions of target proteins may influence which peptides are most readily presented (Figure 4). Additionally, these peptides were presented in spite of the fact that they did not possess optimal binding affinity (Figure 5), and were not necessarily predicted to be the best candidate peptide for MHC-I processing and presentation as analyzed using IEDB prediction tools (Table S7) (42, 43). The lack of a distinct peptide from BRD4 might be due to the fact that the conserved peptide (RLAELQEQL) is the most optimal peptide from the appropriate region of BRD4.

If PROTAC compounds are able to direct the production of MHC-I peptides from specific regions of the targeted protein, then this process could be modulated by the selectivity of the PROTAC-driven ubiquitination process. PROTAC compounds have been shown to result in the ubiquitination of specific protein lysine residues, distinctly from endogenous ubiquitination sites (22). It is accepted that proteasomal processing products drive the MHC-I peptide repertoire through the production of MHC-I peptide precursors (44). Site specific or protein-specific changes in ubiquitination might lead to alterations in proteasomal processing, effecting sequences and availability of MHC-I precursor peptides (44, 45). This hypothesis is supported by the sub-optimal affinities of our identified MHC-I peptides derived from target BET proteins. Understanding the relationship between specific ubiquitination sites and MHC-I peptide presentation is an intriguing future direction.

In addition to BET-derived MHC-I peptides, we observed a number of other peptides induced by PROTAC treatment. Using IPA to map PROTAC-unique and PROTAC-enhanced identifications, we observed an enrichment of both direct and compensatory effects of BET inhibition. Specifically, we observed that Myc was identified to be an upstream regulator of PROTAC-induced identifications. Disruption of the Myc pathway is thought to be the BET inhibitor mechanism of action in cancer (46). Upon degradation of BET proteins, (as opposed to inhibition) others have noted that CDK4/6 and its related pathway are also affected (47). This observation implies that BET inhibition and BET degradation might induce orthogonal modes of cellular regulation, beyond the Myc pathway alone. Our observation of CDK4/6, E2F1, and E2F4 (among others) as potential upstream regulators of PROTAC-induced peptide presentation indicates that these pathway-level changes can be observed within the immunopeptidome.

Taking this information into consideration, we hypothesize that peptides induced or enhanced by PROTAC treatment are derived from proteins that represent three main classes: (1) proteins that degrade due to on-target or off-target interactions with PROTAC compounds; (2) proteins that are directly or indirectly regulated by the PROTAC targeted pathway (bystanders); and (3) general cellular death or stress signals (e.g., glycolysis signaling increased in all treated samples). Among the most-represented PROTAC-specific peptides observed in this study, there remain additional opportunities to gain future biological insights into BET signaling (through inhibition vs. degradation) via MHC-I presentation, as well as, general PROTAC-induced cellular changes, which could reflect the type of E3 ligase engaged and the disruption of their natural function.

We hypothesize that use of PROTAC compounds to induce new targets for immunotherapy will rely on several compounding factors: (1) the pairing between PROTAC and target protein should productively ubiquitinate the target protein resulting in protein degradation, (2) PROTAC-induced degradation should be optimized to slowly to degrade each target, resulting in a longer-lived supply of source peptide for MHC-I processing, and therefore extended presentation of the induced MHC-I peptide, (3) the target protein should be accessible to MHC-I processing and presentation machinery: originating from the compartments and/or biological process that have been empirically observed by us and others as preferred for MHC-I presentation. We also expect that the use of T-cell based therapies should reduce the dependence of target immunogenicity (i.e., even if the induced peptides are not immunogenic, they can still be targeted). Additionally, while we relied on a PROTAC compound to induce the presentation of MHC-I peptides through directed protein degradation, it is possible that any type of treatment that is destabilizing to a protein target (or downstream effector) might suffice to induce the selective presentation of MHC-I peptides.

In conclusion, PROTAC compounds originally emerged as a way to “drug the undruggable” by allowing unprecedented access to proteins and pathways that were previously unavailable due to lack of conventional small molecule inhibition. Here, we have demonstrated a novel capability of PROTAC compounds to induce the presentation of distinct peptides from endogenously expressed target proteins. In addition, we have shown that peptides from non-target source proteins may also be induced for presentation on the cell surface, which opens opportunities to uncover novel protein associations and increase understanding of disease biology. Through using a PROTAC compound to alter the equilibrium of available immunopeptides and induce novel presentation on the cell surface, this strategy may further widen the scope of “druggable space” by offering empirically detectable target options against which T-cell based therapeutics could be designed.

## Data availability

The complete list of identified MHC-I peptides from BV173 cells across all treatments is available in the supplemental information. Flow cytometry data was downloaded into the flow repository ID: FR-FCM-ZYS8.

## Author contributions

Conceptualization: SJ and MP. Methodology: SJ and GP. Investigation: SJ and GP. Data Curation: SJ and DR. Visualization: SJ and DR. Writing Original Draft: SJ. Writing Review and Editing: SJ, MP, GP, and DR. Supervision: MP.

### Conflict of interest statement

The design, study conduct, and financial support for this research were provided by AbbVie. AbbVie participated in the interpretation of data, review, and approval of the publication. The authors declare that the research was conducted in the absence of any commercial or financial relationships that could be construed as a potential conflict of interest.

## Supplementary material

Figure S1 PROTAC compounds induce the degradation of BRD4, BRD3, and BRD2. BV173 cells were treated with 0–1000 nM of each PROTAC for 16 h. Blots were performed against BRD4, BRD3, and BRD2. JQ1-MDM2, JQ1-CRBN, and JQ1-VHL successfully degraded all three BRD proteins. JQ1-VHL and JQ1-MDM2 show modest selectivity between BET family members. JQ1-CRBN does not induce strong degradation at higher concentrations of PROTAC, demonstrating the previously described “hooking effect” characteristic of saturating concentrations of PROTAC (12).

Figure S2 PROTAC compounds show differential degradation of BRD4, BRD3, and BRD2 over a 6 h time course at 10 nM. BV173 cells were treated with 10 nM of each PROTAC for 0–6 h. Western blots were performed against BRD4, BRD3, and BRD2. Within a 6 h timecourse at 10 nM, JQ1-VHL, and JQ1-CRBN were the most successful at degrading target proteins, however, both PROTAC compounds only limitedly degraded BRD2. JQ1-MDM2 did not notably degrade BRD2 within 6 h at 10 nM.

Figure S3 Amount of MHC-I on the cell surface does not change after PROTAC treatment. MHC-I concentration after PROTAC treatment for 0–6 h as measured by FACS, staining with BB7.2 (HLA-A2) and W6/32 (HLA-A/B/C). Median fluorescence intensity (MFI) was plotted as the average of three replicates. Error bars indicate SD.

Figure S4 Affinity of all identified MHC-I peptides, analyzed by ANN (top) and SMM (bottom) algorithms. Affinity (SMM and ANN) mapped for single alleles using the IEDB database (only 4 of 6 BV173 alleles were available in IEDB for affinity mapping). Small shifts in mid-range [Log(IC50) = 2–4] affinity were observed in PROTAC-treated samples vs. DMSO when analyzed by both SMM and ANN algorithms. These changes were specific to HLA-A2 and HLA-B18 alleles.

Figure S5 Proteomaps analysis of BV73 MHC-I Immunopeptidome. Proteomaps provides a visual representation of the MHC-I “regular-ome.” The Proteomaps tool assigns KEGG protein annotations to identified proteins. The area of each polygon is equivalent to the number of unique peptide identifications per source protein, multiplied by protein length. This Proteomap was generated from our complete set of MHC-I peptides, isolated across all treatments and time points. This distribution was not found to vary between analyzed samples.

Figure S6 Matching synthetic MS/MS spectra for identified BRD peptides. Synthetic peptides were used to confirm BRD peptide identification using MS/MS CID fragmentation. All identifications were made with < 5 ppm precursor mass error and ΔRT < 2 min.

Figure S7 Example XIC comparison indicates BRD peptide presentation is unique to PROTAC treatment. The extracted ion chromatogram (XIC) for KMPDEPVEA peptide (m/z = 508.2416, z = 2) from a JQ1-CRBN-treated sample, a JQ1-treated sample, and DMSO- (vehicle) treated sample. Peak extraction was performed with a 10 ppm mass tolerance. XIC AUC = 5^*^10^6^. KMPDEPVEA identification was unique to PROTAC-treated samples.

Figure S8 Housekeeping abundance distributions between samples. Samples produced a range of summed abundances across the raw MS data files acquired, depending on sample to sample IP yield and instrument performance. “Housekeeping” peptides used for normalization in our label free quantification experiments showed a proportionate distribution of individual abundances (XIC AUC) with total (sum) sample abundance.

Figure S9 IPA Canonical Pathway Representation: Protein identifications that were unique to or increased by PROTAC treatment were analyzed by IPA (Qiagen) pathway analysis. Overlapping pathways from the dataset are displayed—increasing color indicates improved *p-*value (*p-*values are listed below each pathway).

Figure S10 IPA Upstream Regulator Prediction: Protein identifications that were unique to or increased by PROTAC treatment were analyzed by IPA (Qiagen) pathway analysis. Upstream regulators (white) of observed proteins/genes (gray) are depicted, the *p-*values determined are the following: ERBB2, 2.83E-03; CST5, 2.08E-03; E2F1, 7.82E-04; MYC, 1.29E-02; CCND1, 6.44E-03; E2F4, 1.16E-03; IL15, 7.04E-04; IL13, 3.81E-02; CDK4/6, 6.32E-04.

Table S1 All observed HLA peptides.

Table S2 Complete list of changes to GO protein annotations for cellular compartment and biological processes using Panther database separated by treatment type.

Table S3 All identified PROTAC-specific unique source proteins (shared between all 3 PROTAC treatments). These proteins are ranked by number of PSMs.

Table S4 Related to Figures 3C,D.All shared source protein identifications, with fold change values for each PROTAC treatment individually and as an average. Comparing proteins observed in both treated and untreated samples only.

Table S5 Extracted ion chromatogram area under the curve (XIC AUC) values and replicate information for the peptides observed in Figures 6, 7.

Table S6 Housekeeping peptide list: 29 peptides and source proteins used for sample abundance (XIC AUC) normalization.

Table S7 IEDB MHC-I processing prediction analysis for BRD2, BRD3, and BRD4 individually (HLA-A^*^02:01 predictions only).
