# Supplementary material for: Specific MHC-I Peptides Are Induced Using PROTACs
Source: Front Immunol. 2018 Nov 22;9:2697. doi: 10.3389/fimmu.2018.02697 (PMC6262898; doi:10.3389/fimmu.2018.02697)
Supplement: Figure S1 — PROTAC compounds induce the degradation of BRD4, BRD3, and BRD2. BV173 cells were treated with 0–1000 nM of each PROTAC for 16 h. Blots were performed against BRD4, BRD3, and BRD2. JQ1-MDM2, JQ1-CRBN, and JQ1-VHL successfully degraded all three BRD proteins. JQ1-VHL and JQ1-MDM2 show modest selectivity between BET family members. JQ1-CRBN does not induce strong degradation at higher concentrations of PROTAC, demonstrating the previously described “hooking effect” characteristic of saturating concentrations of PROTAC (12). [file Data_Sheet_1.pdf]

Supplementary Material

Specific MHC-I Peptides are Induced using PROTACs

Authors:

Stephanie M. Jensen, Gregory K. Potts, Damien B. Ready, and Melanie J. Patterson[*]

Correspondence:

Melanie.Patterson@abbvie.com

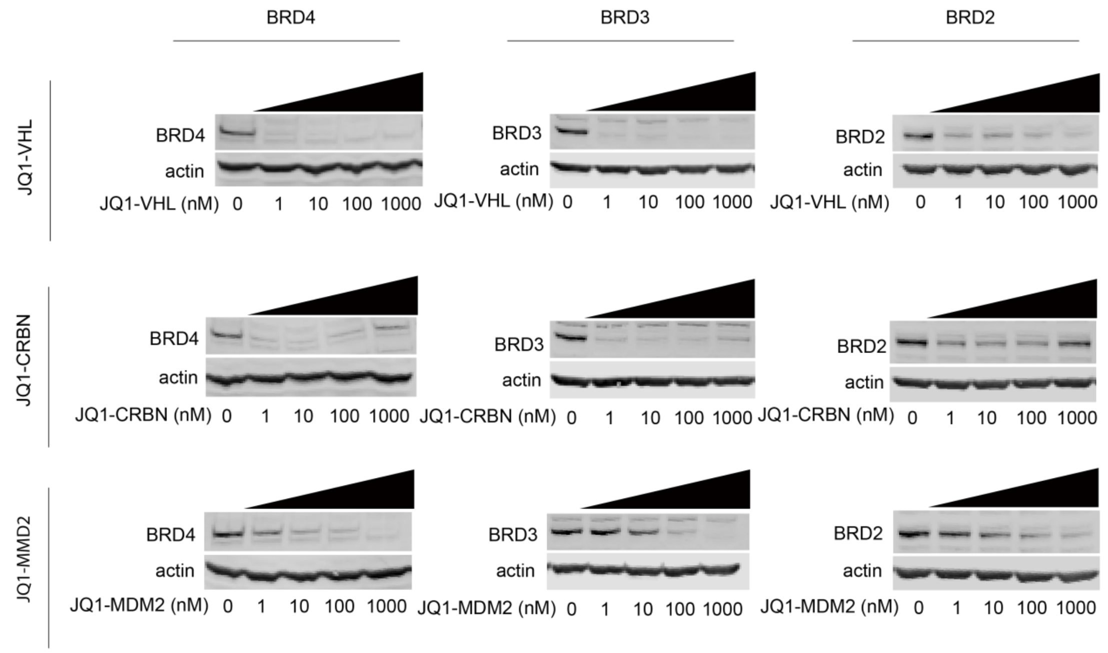

Figure S1. PROTAC compounds induce the degradation of BRD4, BRD3, and BRD2. BV173 cells were treated with 0-1000 nM of each PROTAC for 16 hours. Blots were performed against BRD4, BRD3, and BRD2. JQ1-MDM2, JQ1-CRBN, and JQ1-VHL successfully degraded all three BRD proteins. JQ1-VHL and JQ1-MDM2 show modest selectivity between BET family members. JQ1-CRBN does not induce strong degradation at higher concentrations of PROTAC, demonstrating the previously described "hooking effect" characteristic of saturating concentrations of PROTAC (Huang and Dixit, 2016).

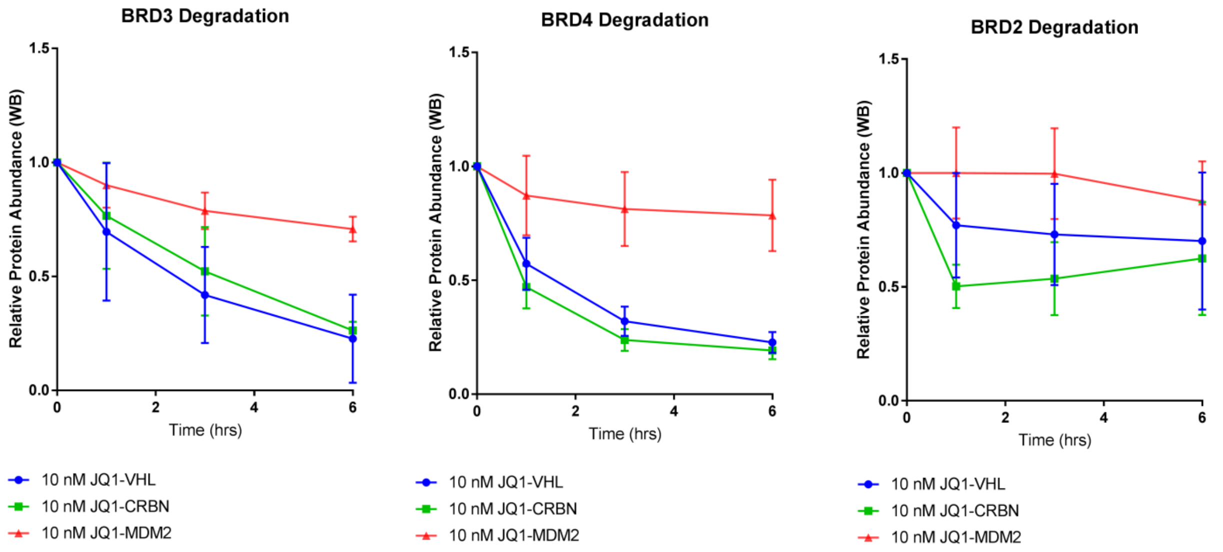

Figure S2. PROTAC compounds show differential degradation of BRD4, BRD3, and BRD2 over a 6 hour time course at 10 nM. BV173 cells were treated with 10 nM of each PROTAC for 0-6 hours. Western blots were performed against BRD4, BRD3, and BRD2. Within a 6 hour timecourse at 10 nM, JQ1-VHL and JQ1-CRBN were the most successful at degrading target proteins, however, both PROTAC compounds only limitedly degraded BRD2. JQ1-MDM2 did not notably degrade BRD2 within 6 hours at 10 nM.

Figure S

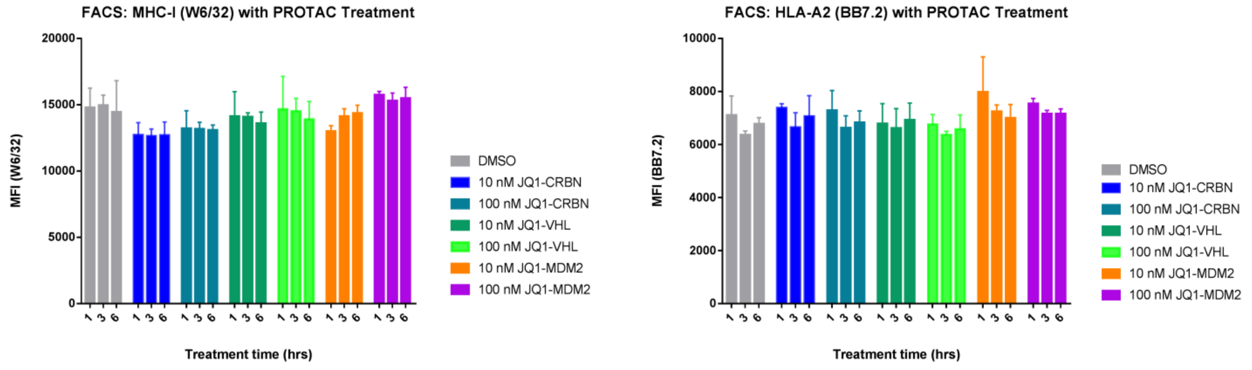

Figure S3. Amount of MHC-I on the cell surface does not change after PROTAC treatment. MHC-I concentration after PROTAC treatment for 0-6 hours as measured by FACS, staining with BB7.2 (HLA-A2) and W6/32 (HLA-A,B,C). Median fluorescence intensity (MFI) was plotted as the average of three replicates. Error bars indicate SD.

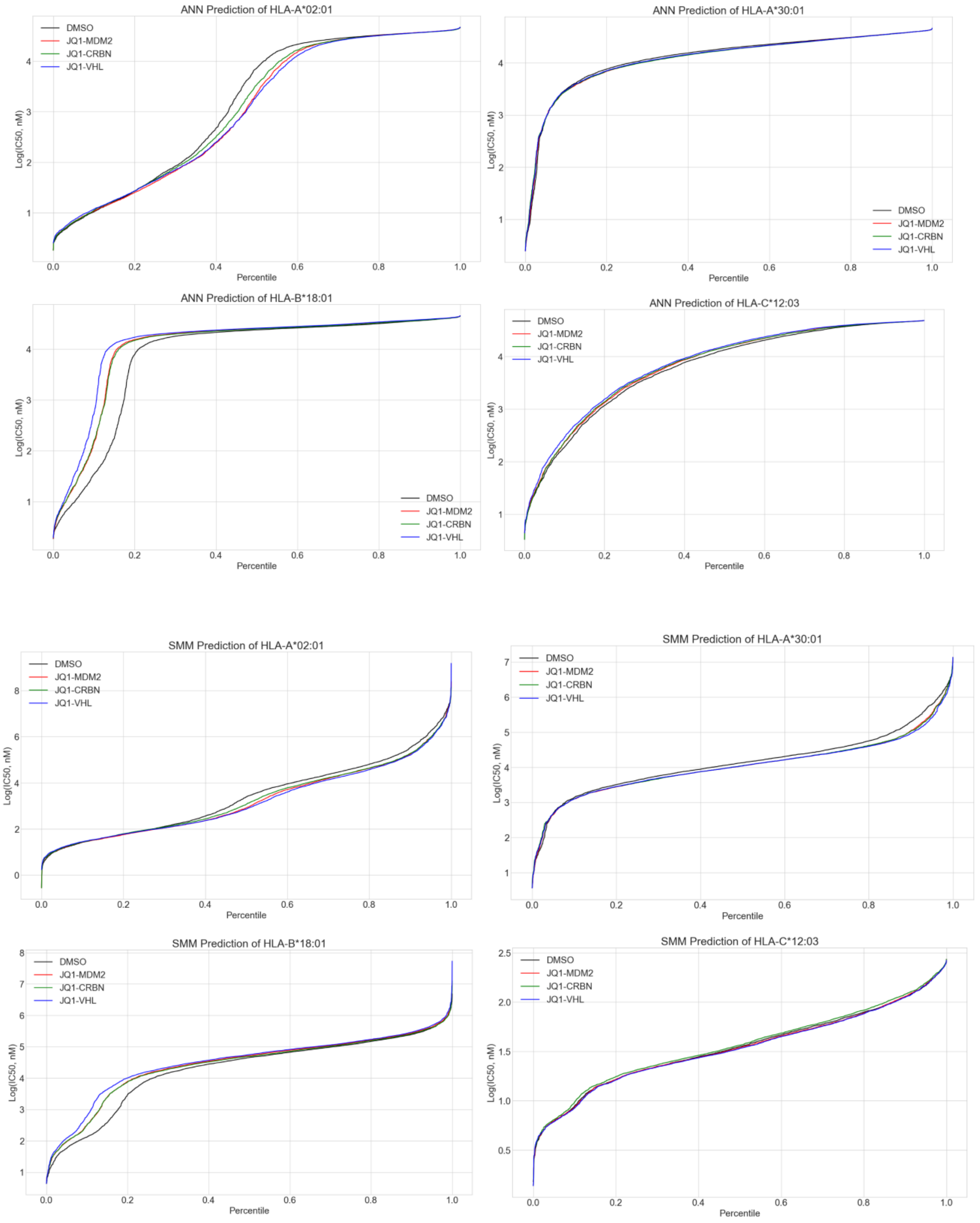

Figure S4. Affinity of all identified MHC-I peptides, analyzed by ANN (top) and SMM (bottom) algorithms. Affinity (SMM and ANN) mapped for single alleles using the IEDB database (only 4 of 6 BV173 alleles were available in IEDB for affinity mapping). Small shifts in mid-range (Log(IC50) = 2-4) affinity were observed in PROTAC-treated samples versus DMSO when analyzed by both SMM and ANN algorithms. These changes were specific to HLA-A2 and HLA-B18 alleles.

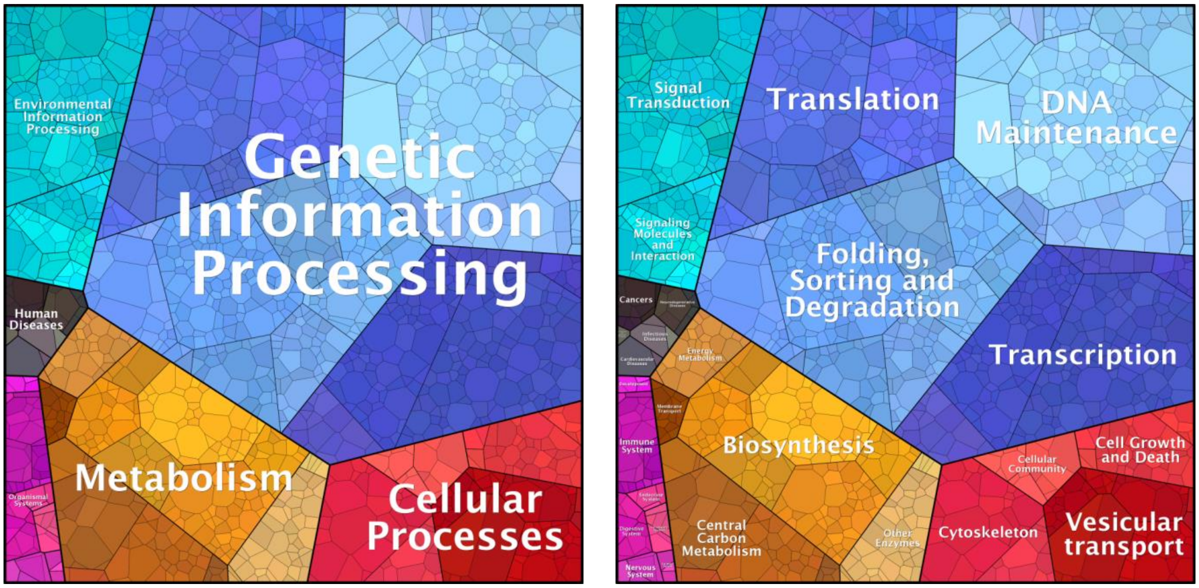

Figure S5: Proteomaps analysis of BV73 MHC-I Immunopeptidome. Proteomaps provides a visual representation of the MHC-I "regular-ome". The Proteomaps tool assigns KEGG protein annotations to identified proteins. The area of each polygon is equivalent to the number of unique peptide identifications per source protein, multiplied by protein length. This Proteomap was generated from our complete set of MHC-I peptides, isolated across all treatments and time points. This distribution was not found to vary between analyzed samples.

HQVPAVSSV (BRD2)

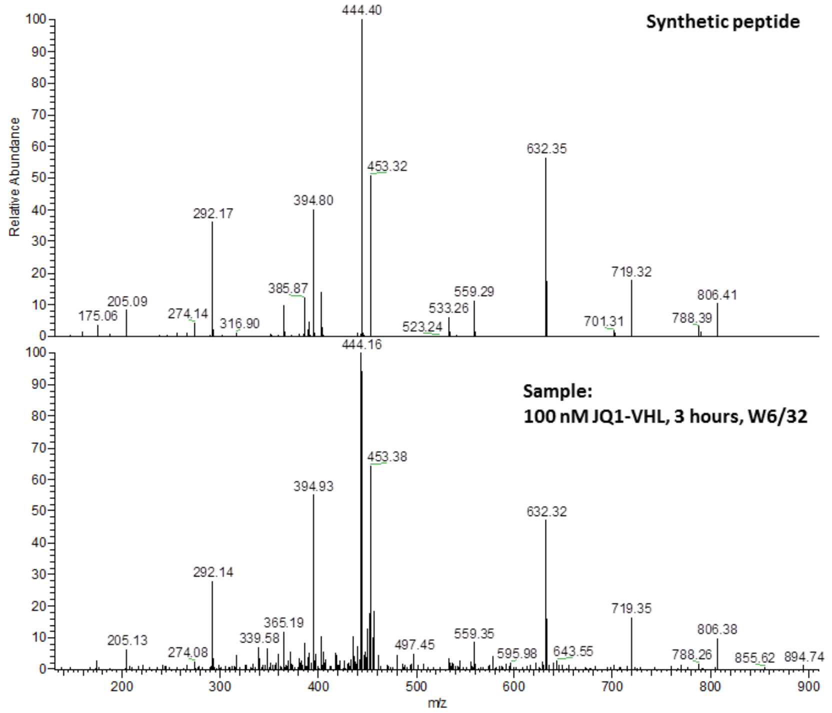

KMPDEPVEA (BRD3)

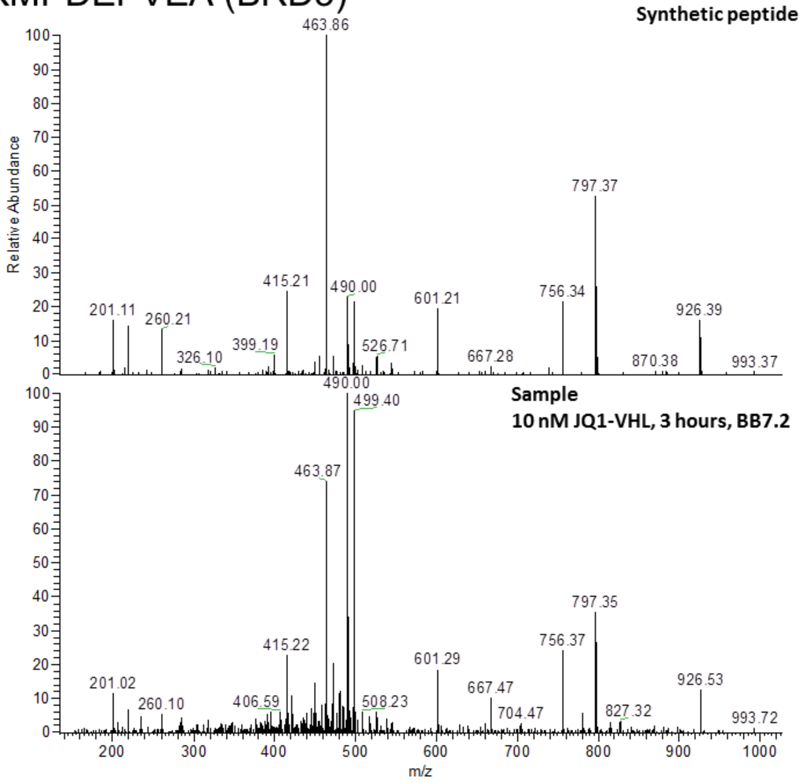

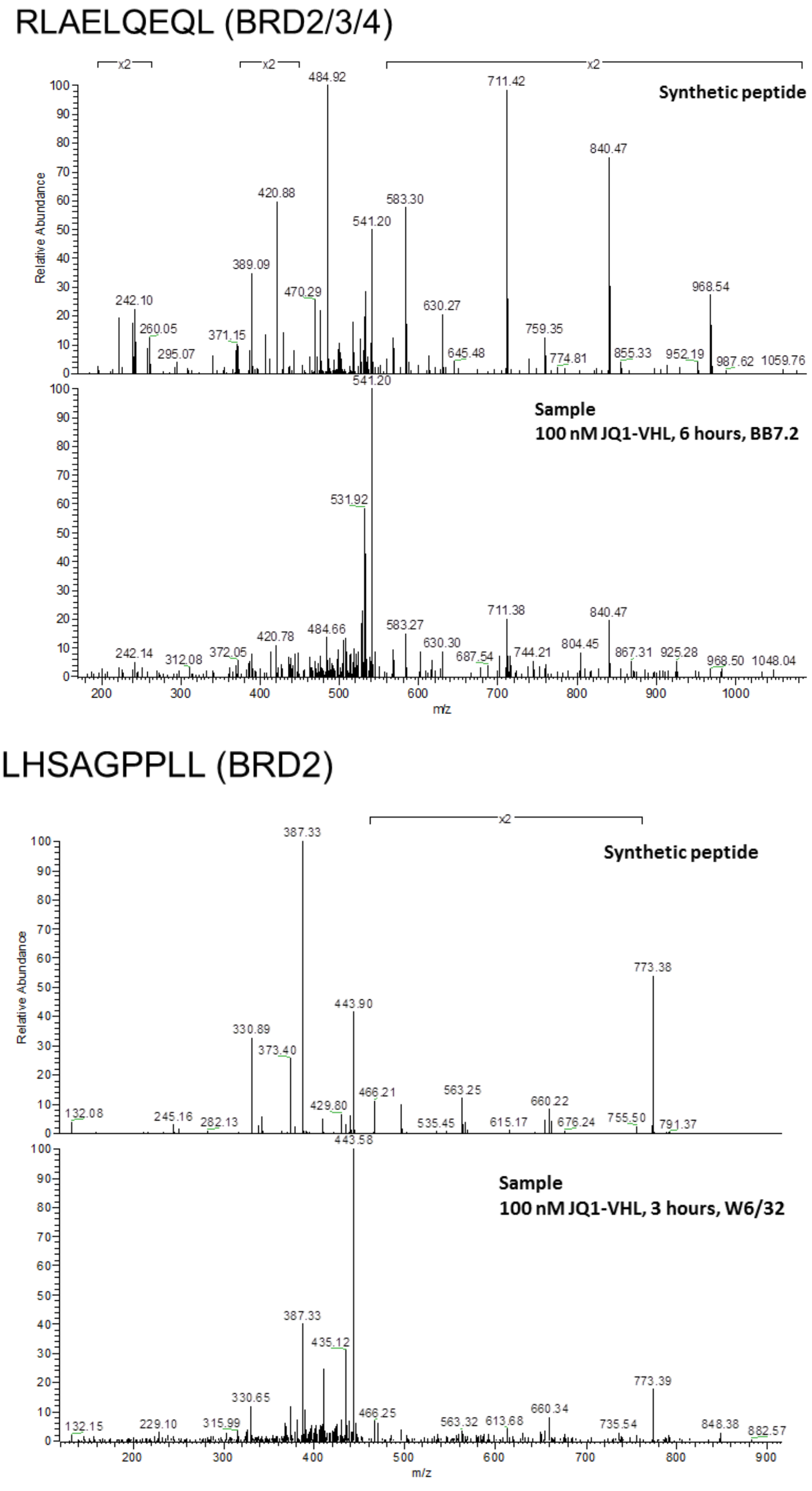

Figure S6. Matching synthetic MS/MS spectra for identified BRD peptides. Synthetic peptides were used to confirm BRD peptide identification using MS/MS CID fragmentation. All identifications were made with <5 ppm precursor mass error and ΔRT <2 minutes.

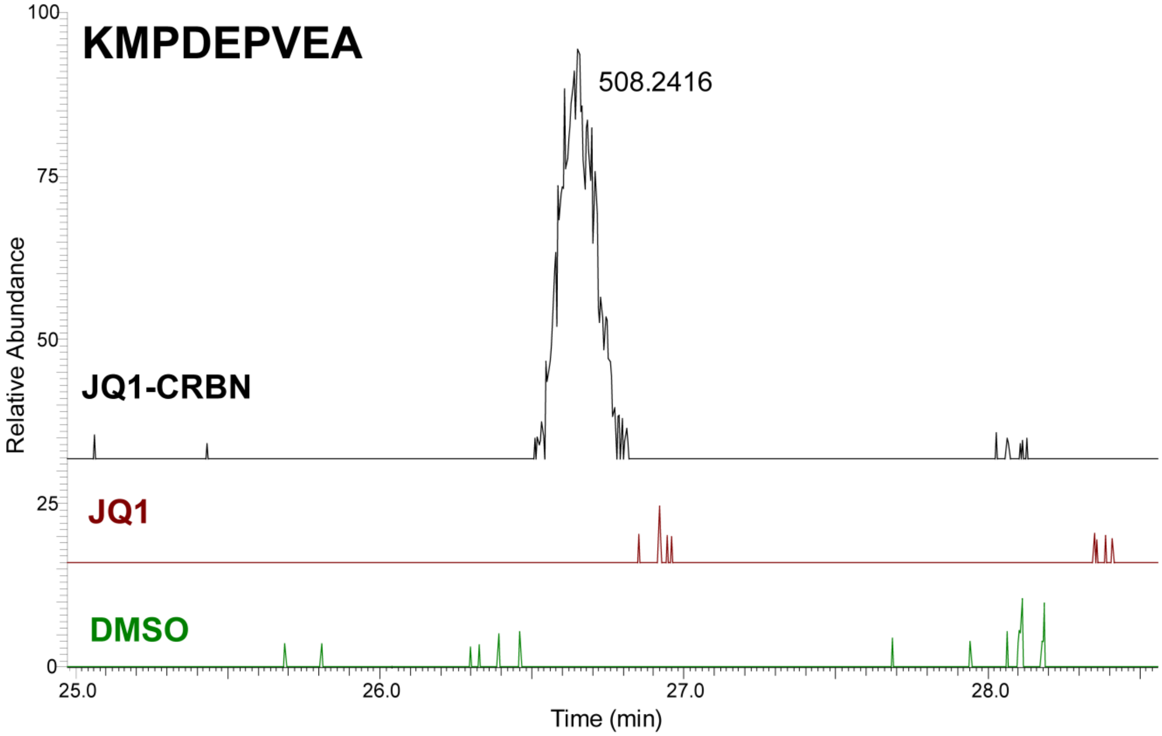

Figure S7. Example XIC comparison indicates BRD peptide presentation is unique to PROTAC treatment. The extracted ion chromatogram (XIC) for KMPDEPVEA peptide (m/z=508.2416, z=2) from a JQ1-CRBN-treated sample, a JQ1-treated sample, and DMSO- (vehicle) treated sample. Peak extraction was performed with a 10 ppm mass tolerance. XIC AUC = $5*10^6$. KMPDEPVEA identification was unique to PROTAC-treated samples.

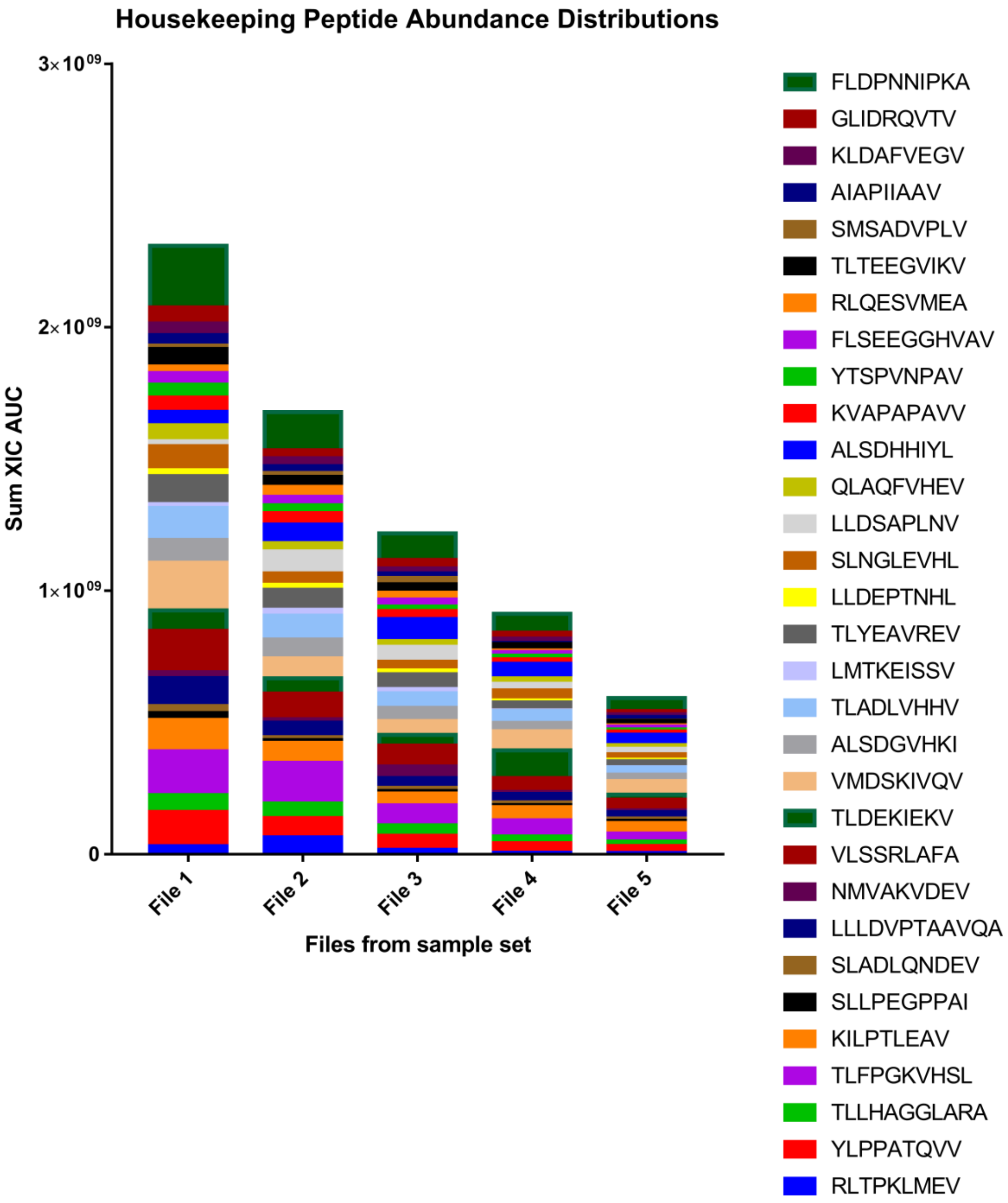

Figure S8. Housekeeping abundance distributions between samples. Samples produced a range of summed abundances across the raw MS data files acquired, depending on sample to sample IP yield and instrument performance. "Housekeeping" peptides used for normalization in our label free quantification experiments showed a proportionate distribution of individual abundances (XIC AUC) with total (sum) sample abundance.

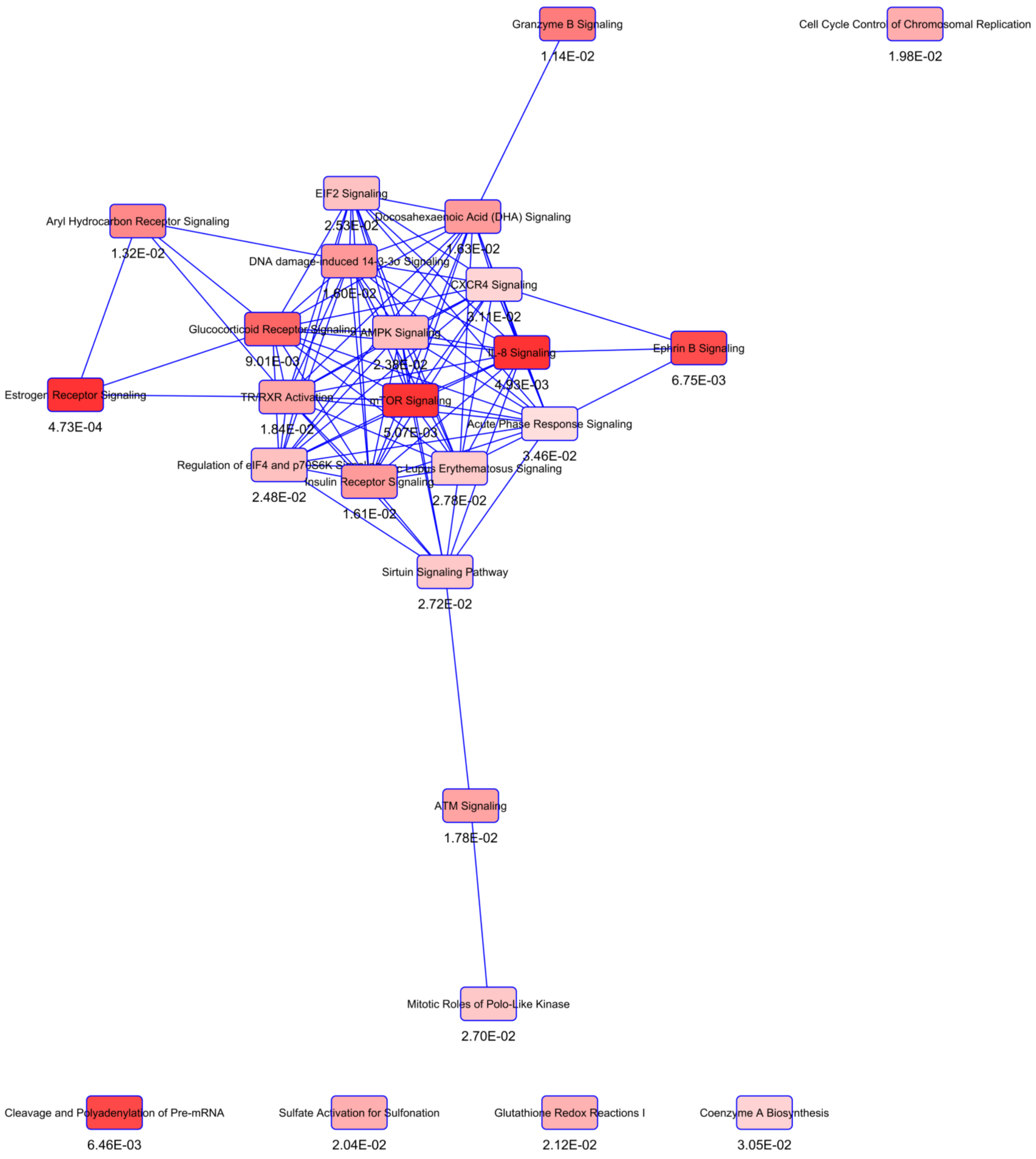

© 2000-2018 QIAGEN. All rights reserved.

Figure S9. IPA Canonical Pathway Representation: Protein identifications that were unique to or increased by PROTAC treatment were analyzed by IPA (Qiagen) pathway analysis. Overlapping pathways from the dataset are displayed - increasing color indicates improved *p*-value (*p*-values are listed below each pathway).

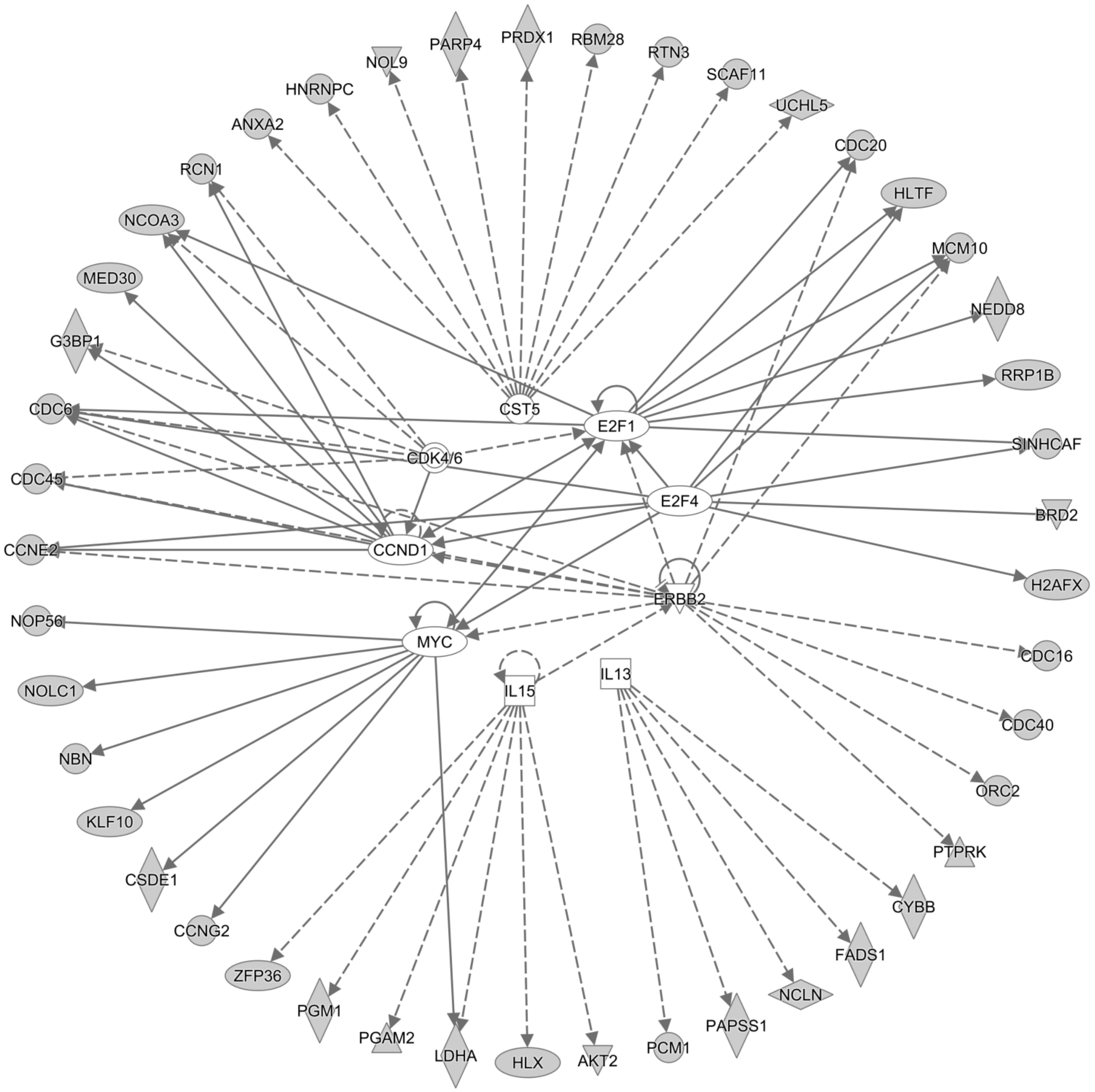

© 2000-2018 QIAGEN. All rights reserved.

Figure S10. IPA Upstream Regulator Prediction: Protein identifications that were unique to or increased by PROTAC treatment were analyzed by IPA (Qiagen) pathway analysis. Upstream regulators (white) of observed proteins/genes (gray) are depicted, the *p*-values determined are the following: ERBB2, 2.83E-03; CST5, 2.08E-03; E2F1, 7.82E-04; MYC, 1.29E-02; CCND1, 6.44E-03; E2F4, 1.16E-03; IL15, 7.04E-04; IL13, 3.81E-02; CDK4/6, 6.32E-04.
