# Supplementary material for: Specific MHC-I Peptides Are Induced Using PROTACs
Source: Front Immunol. 2018 Nov 22;9:2697. doi: 10.3389/fimmu.2018.02697 (PMC6262898; doi:10.3389/fimmu.2018.02697)
Supplement: Table S6 — Housekeeping peptide list: 29 peptides and source proteins used for sample abundance (XIC AUC) normalization. [file Table_6.pdf]

Sequence	Accession	Gene Name	Description
RLTPKLMEV	O15372	EIF3H	Eukaryotic translation initiation factor 3 subunit H
YLPPATQVV	P38919	EIF4A3	Eukaryotic initiation factor 4A-III
TLLHAGGLARA	P0C7U0	ELFN1	Protein ELFN1
TLFPGKVHSL	Q9NNW5	WDR6	WD repeat-containing protein 6
KILPTLEAV	Q12905	ILF2	Interleukin enhancer-binding factor 2
SLLPEGPPAI	Q15011	HERPUD1	Homocysteine-responsive endoplasmic reticulum-resident ubiquitin-like domain member 1 protein
SLADLQNDEV	P61247	RPS3A	40S ribosomal protein S3a
LLLDVPTAAVQA	P13284	IFI30	Gamma-interferon-inducible lysosomal thiol reductase
NMVAKVDEV	P62906	RPL10A	60S ribosomal protein L10a
VLSSRLAFA	P79483	HLA-DRB3	HLA class II histocompatibility antigen, DR beta 3 chain
TLDEKIEKV	Q96GQ7	DDX27	Probable ATP-dependent RNA helicase DDX27
VMDSKIVQV	O15131	KPNA5	Importin subunit alpha-6
ALSDGVHKI	Q9NVQ4	FAIM	Fas apoptotic inhibitory molecule 1
TLADLVHHV	Q9Y4A5	TRRAP	Transformation/transcription domain-associated protein
LMTKEISSV	P78527	PRKDC	DNA-dependent protein kinase catalytic subunit
TLYEAVREV	P62906	RPL10A	60S ribosomal protein L10a
LLDEPTNHL	Q9UG63	ABCF2	ATP-binding cassette sub-family F member 2
SLNGLEVHL	Q9NQG7	HPS4	Hermansky-Pudlak syndrome 4 protein
QLAQFVHEV	A8MPP1	DDX11L8	Putative ATP-dependent RNA helicase DDX11-like protein 8
ALSDHHIYL	P04075	ALDOA	Fructose-bisphosphate aldolase A
KVAPAPAVV	P62424	RPL7A	60S ribosomal protein L7a
YTSPVNPAV	P61165	TMEM258	Transmembrane protein 258
FLSEEGGHVAV	Q16877	PFKFB4	6-phosphofructo-2-kinase/fructose-2,6-bisphosphatase 4
RLQESVMEA	O60216	RAD21	Double-strand-break repair protein rad21 homolog
TLTEEGVIKV	Q9BZE4	GTPBP4	Nucleolar GTP-binding protein 1
SMSADVPLV	P12004	PCNA	Proliferating cell nuclear antigen
KLDAFVEGV	P21283	ATP6V1C1	V-type proton ATPase subunit C 1
GLIDRQVTV	Q9UPN3	MACF1	Microtubule-actin cross-linking factor 1, isoforms 1/2/3/5
FLDPNNIPKA	Q9BVK2	ALG8	Probable dolichyl pyrophosphate Glc1Man9GlcNAc2 alpha-1,3-glucosyltransferase

Table S6. Housekeeping peptide list: 29 peptides and source proteins used for sample abundance (XIC AUC) normalization.
